# Supplementary material for: Characterising information gains and losses when collecting multiple epidemic model outputs
Source: Epidemics. 2024 Jun;47:100765. doi: 10.1016/j.epidem.2024.100765 (PMC11196924; doi:10.1016/j.epidem.2024.100765)
Supplement: Supplementary file 1 — Supplementary material [file mmc1.pdf]

Supplementary Information

SI Figure 1

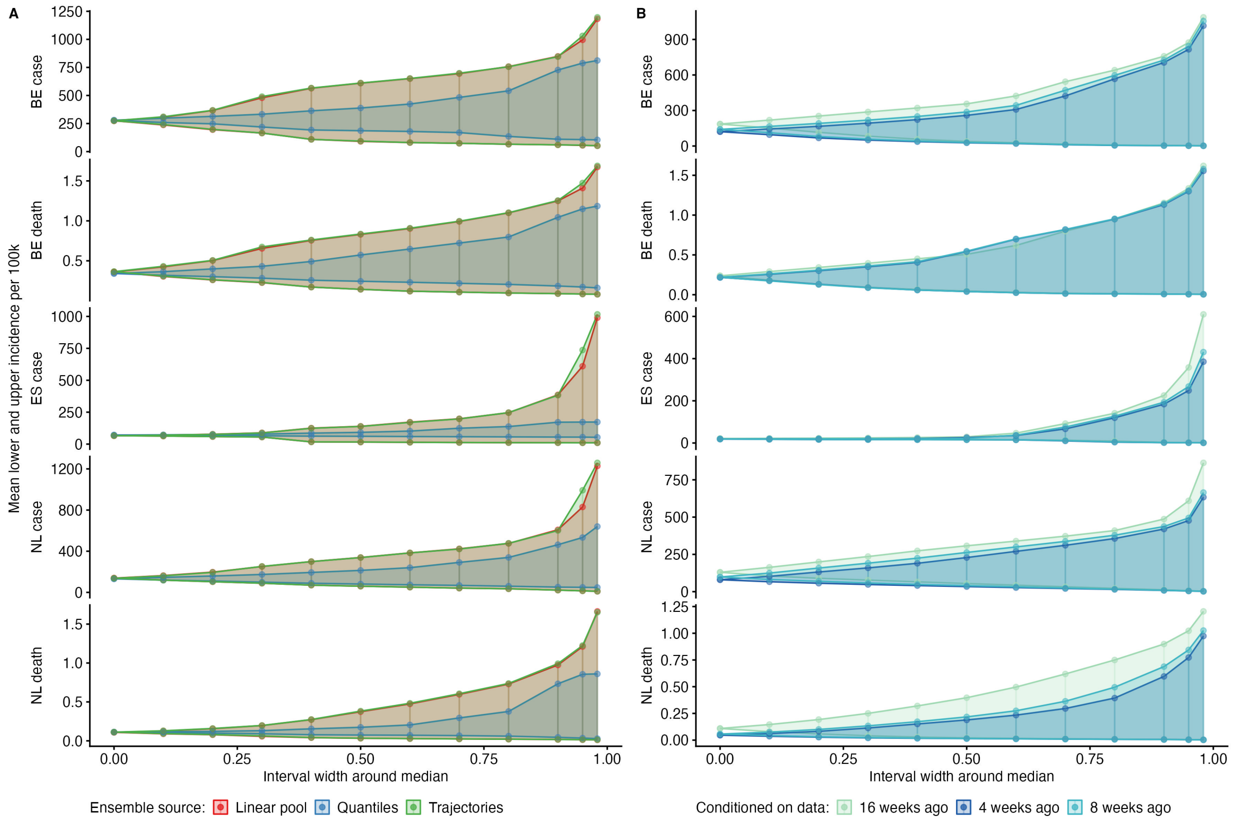

SI Figure 1. Mean central prediction intervals at increasing distances from the median. The 52-week mean of incidence per 100,000 population across all time points and scenarios, showing mean central prediction intervals at increasing distances from the median (interval width), by aggregation method (A) or weighting (B). The median estimate for each ensemble has 0 interval width (x-axis), with uncertainty increasing until an interval width at 0.98 represents the 1%-99% credibility interval around the median.

SI Figure 2

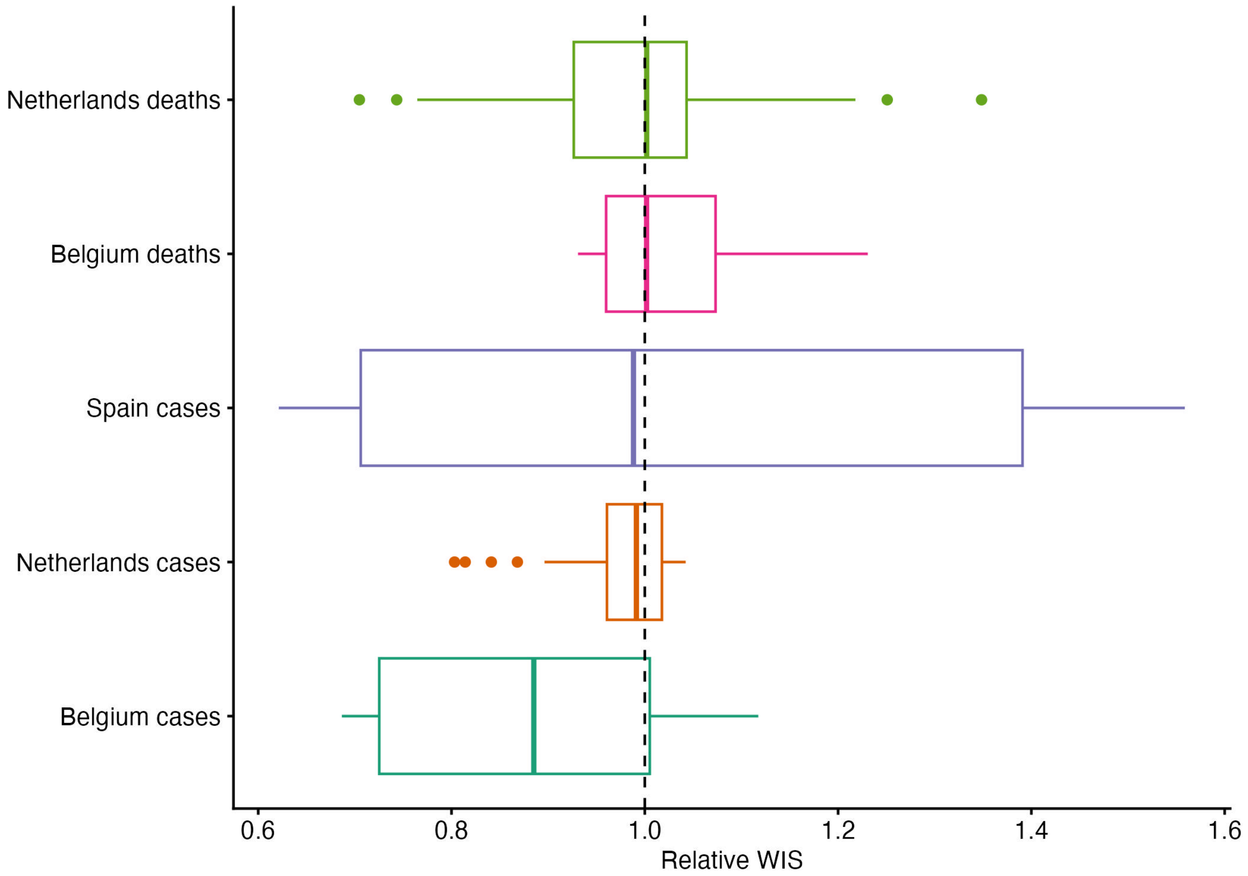

SI Figure 2. Distribution of forecast performance scores (relative WIS), of forecasts from model trajectories weighted using 0 through 31 weeks' available data. Performance is compared to an unweighted ensemble (reference line at 1).

SI Table 1

Team	Methods
**ECDC**	
ECDC-CM_ONE	Discrete-time, deterministic, mean-field SEIR-type compartmental model on metapopulation level. Population divided by age, vaccination status, and previous recovery; incl. seasonality, BA2 & behavior.
**Dutch National Institute of Public Health and the Environment (RIVM)**	
RIVM-vacamole	Deterministic, age-structured SEIR model, accounting for differences in susceptibility/infectiousness by age, seasonality, contact patterns, modes of vaccine protection, and waning immunity.
**SIMID**	
SIMID-SCM	Stochastic age-structured discrete time extended compartmental model
**Universidad Carlos III de Madrid**	

Team	Methods
UC3M-EpiGraph	Agent-based parallel simulator that models individual interactions extracted from social networks and demographical data.
**University of Southern California**	
USC-SIkJalpha	Uses SIKJalpha which models temporally varying infection, death, and hospitalization rates. Learning is performed by reducing the problem to multiple simple linear regression problems.

SI Table 1. Teams that contributed models to Round 2 of the European Scenario Hub, with self-described methods and links to further information. See also:

- Full model metadata, at: https://github.com/covid19-forecast-hub-europe/covid19-scenario-hub-europe/tree/main/model-metadata
- Information about each model's assumptions for Round 2, at: https://github.com/covid19-forecast-hub-europe/covid19-scenario-hub-europe/tree/main/model-abstracts/2022-07-24

Round 2 report

The following pages are the original website reporting for the European Scenario Hub Round 2 as of July 2022.

The report is currently (January 2023) available at: https://covid19scenariohub.eu/report2.html

Code to generate this report is available at: https://github.com/european-modelling-hubs/covid19-scenario-hub-europe-website/blob/main/report2.Rmd

Round 2

Scenarios

We asked teams of researchers across Europe to use quantitative models to project COVID-19 outcomes for 32 European countries over the next year. In order to explore different sets of assumptions about drivers of the pandemic, we asked teams to vary four sets of parameters. We can describe this in a 2x2 scenario specification:

	**Age 60+ booster campaign**<br>• 2nd* booster recommended for 60+<br>• Uptake starts 15th September, and reaches 50% coverage by 15th December	**Age 18+ booster campaign**<br>• 2nd* booster recommended for general population, ages 18+<br>• Uptake starts 15th September, and reaches 50% coverage by 15th December
**Optimistic vaccine effectiveness**<br>• Increased booster vaccine effectiveness to that seen against **Delta variant**	Scenario A	Scenario B
**Pessimistic vaccine effectiveness**<br>• Reduced booster vaccine effectiveness against infection from **BA.4/BA.5/BA.2.75 variants**	Scenario C	Scenario D

See also the full scenario details (https://github.com/covid19-forecast-hub-europe/covid19-scenario-hub-europe/wiki/Round-2) for more detail on the common set of assumptions teams used to create their models.

In Round 2, we asked modellers to start their projections from the 2022-07-24. Data after this date were not included, and as a result, model projections are unlikely to fully account for later information on the changing variants or behavioural patterns.

In this report we only show results from countries with at least 3 models.

Current situation

We consider vaccination rates in countries for which multiple teams of modellers contributed projections.

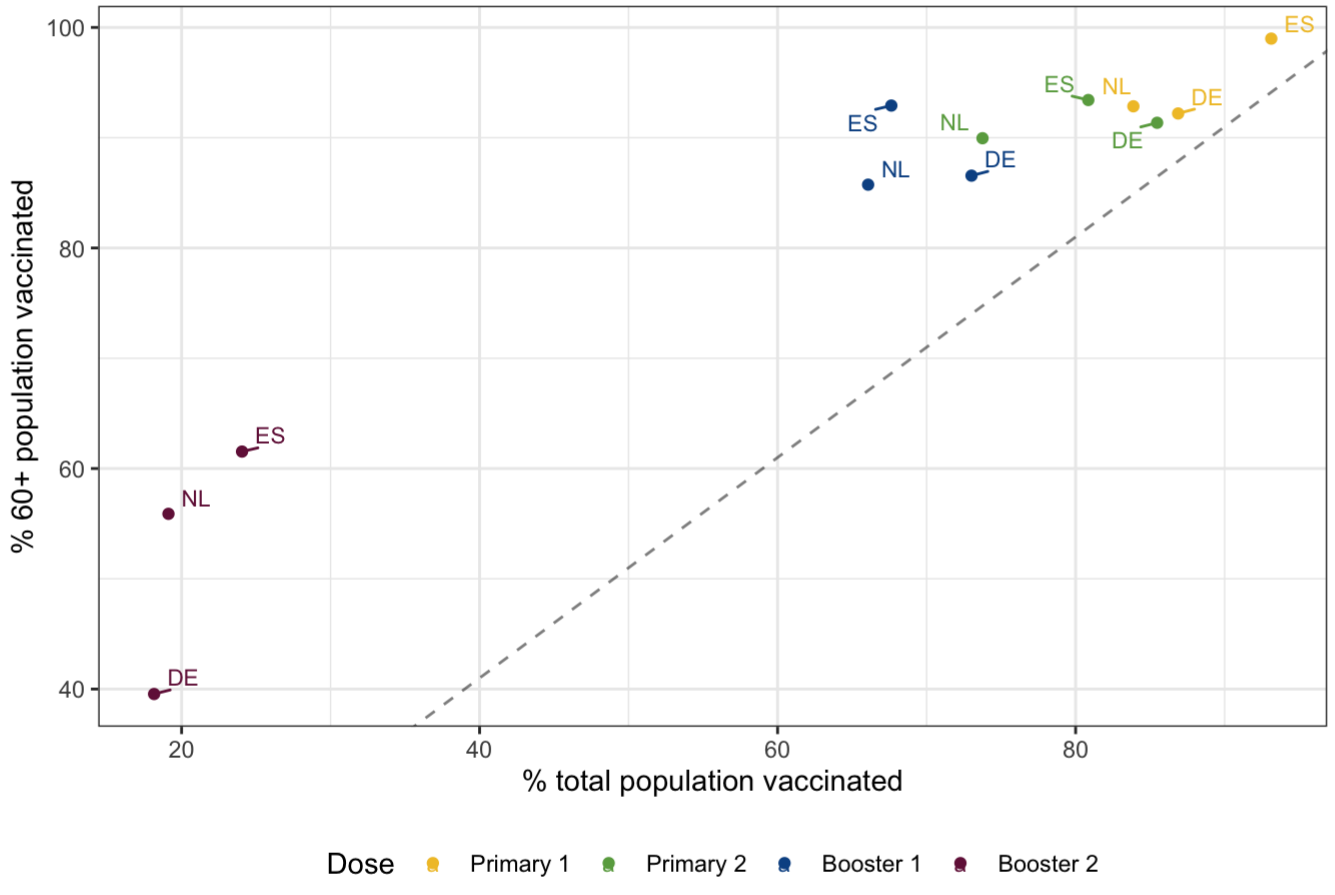

Vaccination % among 60+ population, relative to total population

Participating teams

6 models contributed scenario projections to Round 2.

Models

Participating teams by number of countries and horizon

Team	Countries	Weeks
USC-SIkJalpha	31	52
ECDC-CM_ONE	28	53
MODUS_Covid-Episim	1	53
RIVM-vacamole	1	53
SIMID-SCM	1	52
UC3M-EpiGraph	1	41

Countries

Number of independent model projections for each target variable and location

Code	Country	Infection	Case	Hosp	Icu	Death
BE	Belgium	1	3	2	1	3
DE	Germany	1	2	2	0	1
ES	Spain	1	3	2	0	2

Code	Country	Infection	Case	Hosp	Icu	Death
NL	Netherlands	1	3	2	1	3

Cumulative outcomes

For each model and scenario, we compare the total number of outcomes over the entire projection period as a % of the total country population. We compared the cumulative number of projected outcomes to the cumulative total over one year before projections started (July 2021 to July 2022).

Death

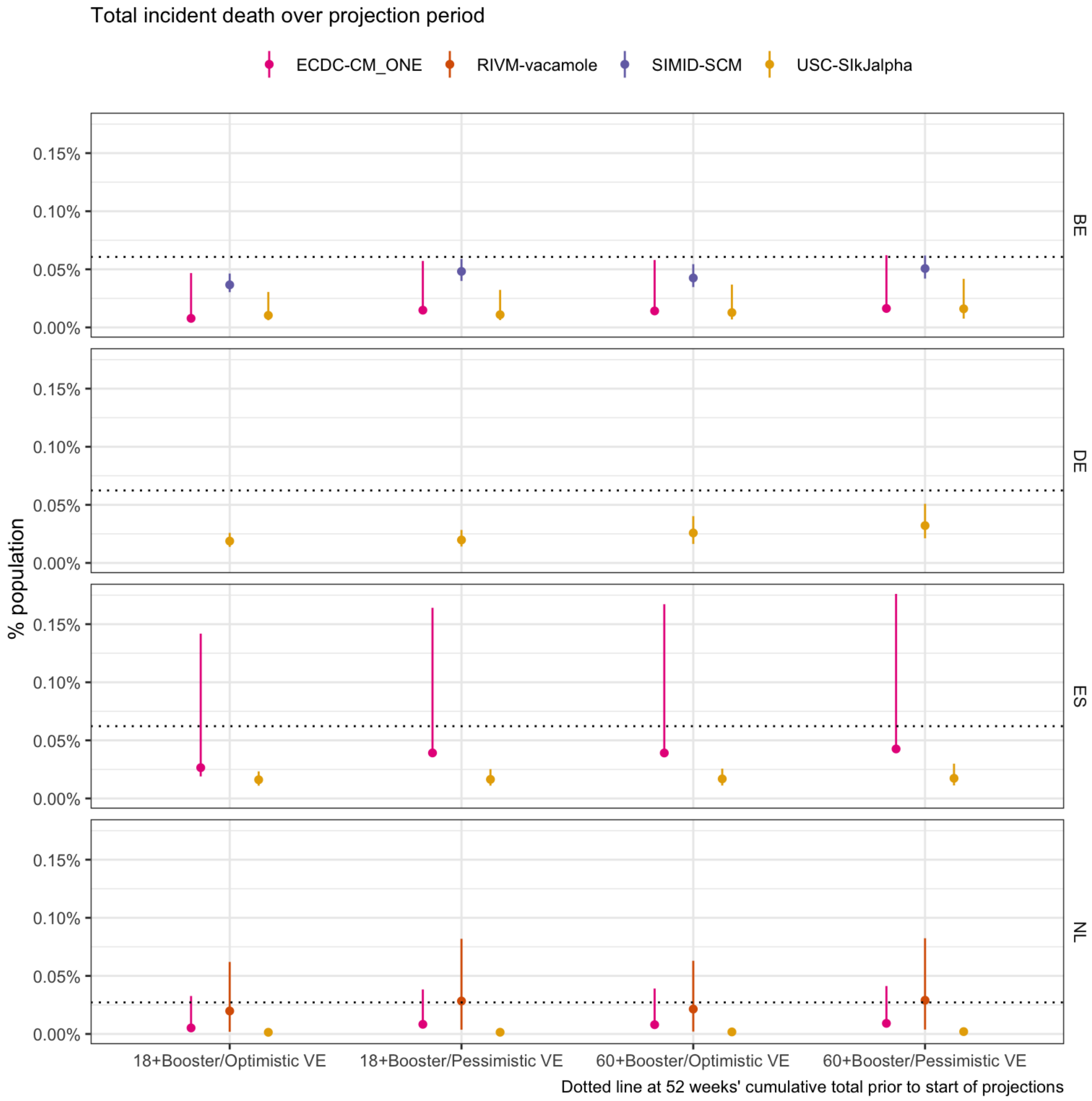

Scenarios: Autumn second booster campaign among population aged ‘18+’ or ‘60+’; Vaccine effectiveness is ‘optimistic’(effectiveness as of a booster vaccine against Delta) or ‘pessimistic’ (as against BA.4/BA.5/BA.2.75)

Case

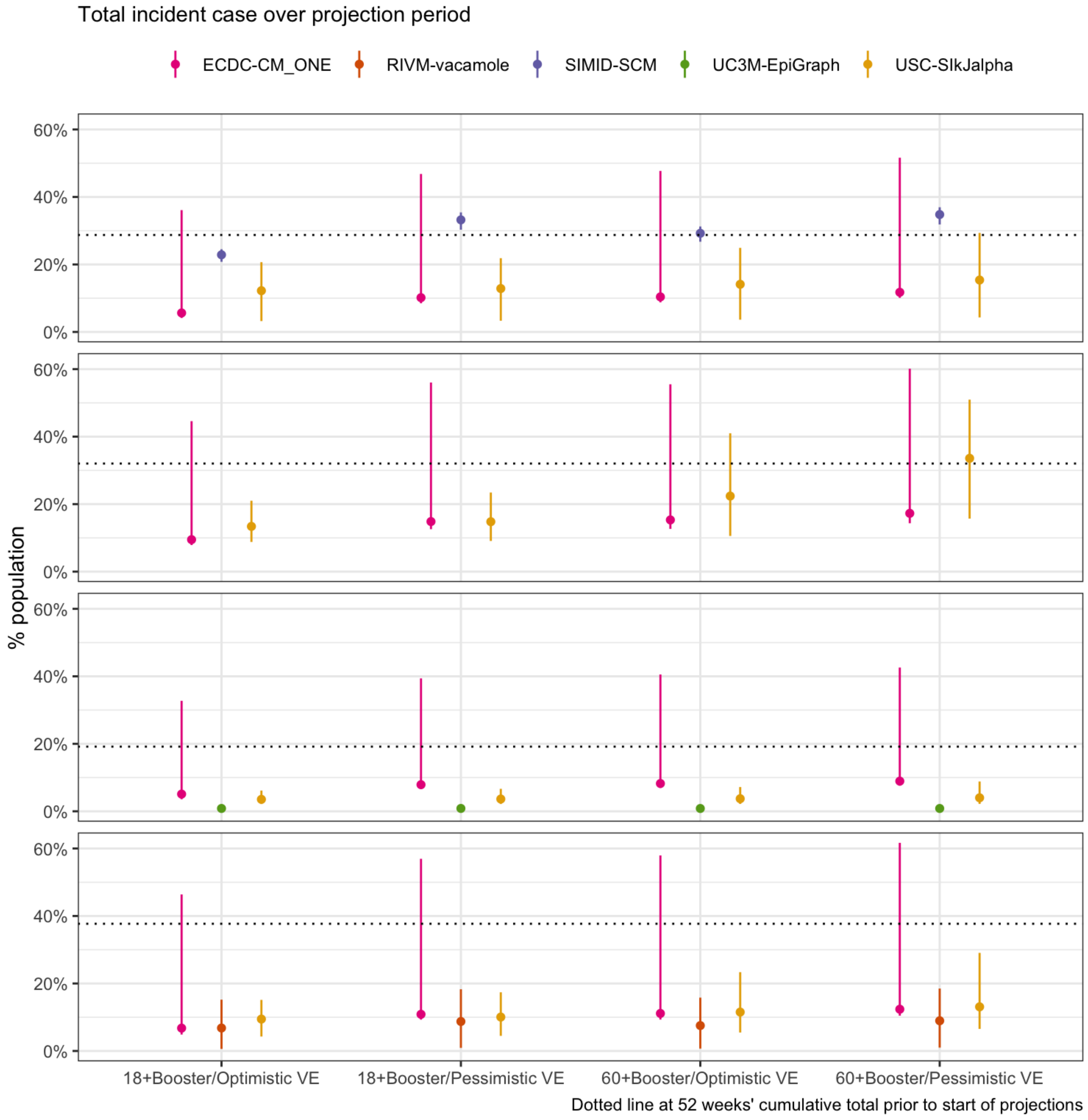

Scenarios: Autumn second booster campaign among population aged ‘18+’ or ‘60+’; Vaccine effectiveness is ‘optimistic’(effectiveness as of a booster vaccine against Delta) or ‘pessimistic’ (as against BA.4/BA.5/BA.2.75)

Infection

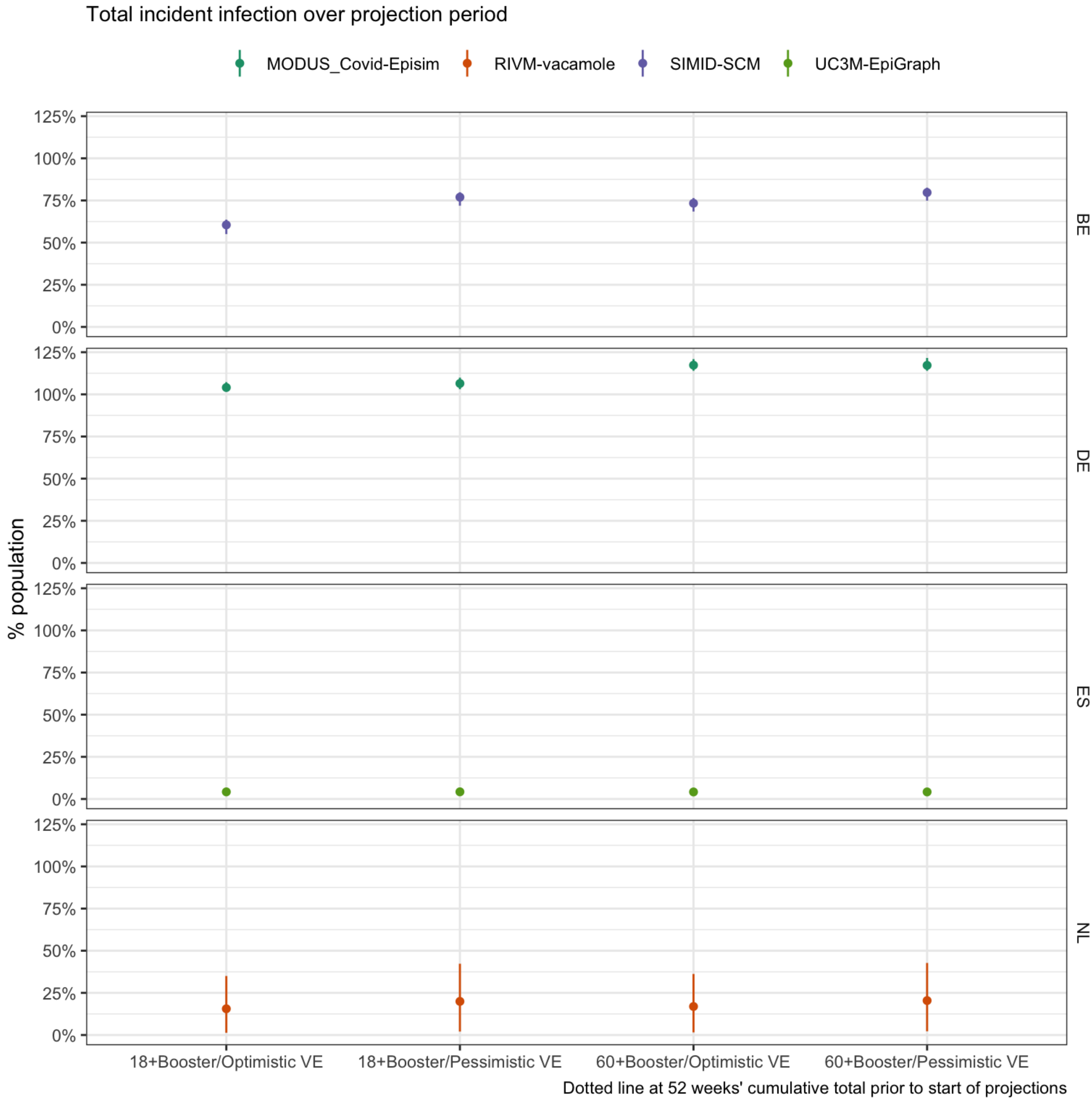

Scenarios: Autumn second booster campaign among population aged ‘18+’ or ‘60+’; Vaccine effectiveness is ‘optimistic’(effectiveness as of a booster vaccine against Delta) or ‘pessimistic’ (as against BA.4/BA.5/BA.2.75)

Hosp

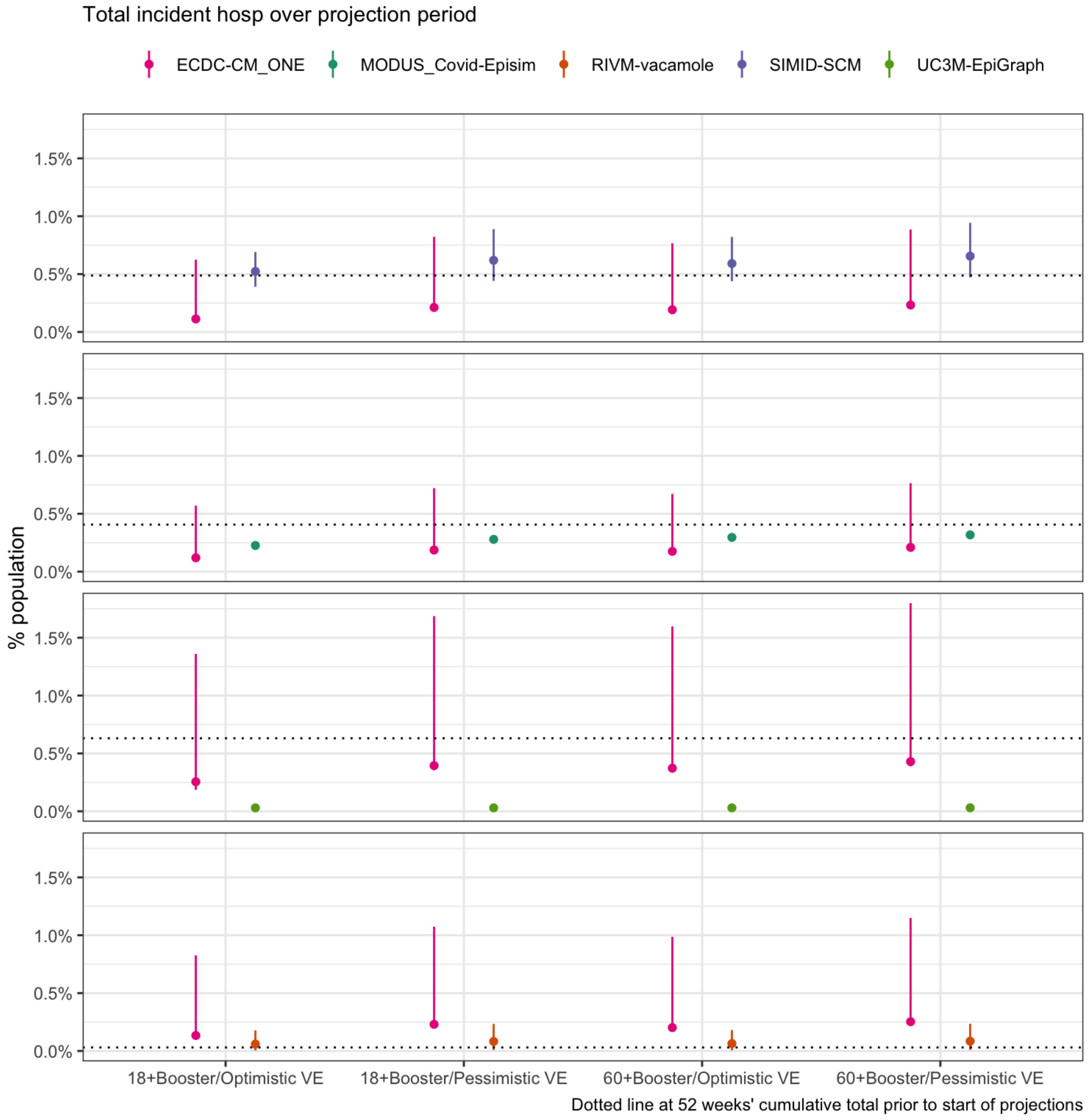

Scenarios: Autumn second booster campaign among population aged ‘18+’ or ‘60+’; Vaccine effectiveness is ‘optimistic’(effectiveness as of a booster vaccine against Delta) or ‘pessimistic’ (as against BA.4/BA.5/BA.2.75)

Icu

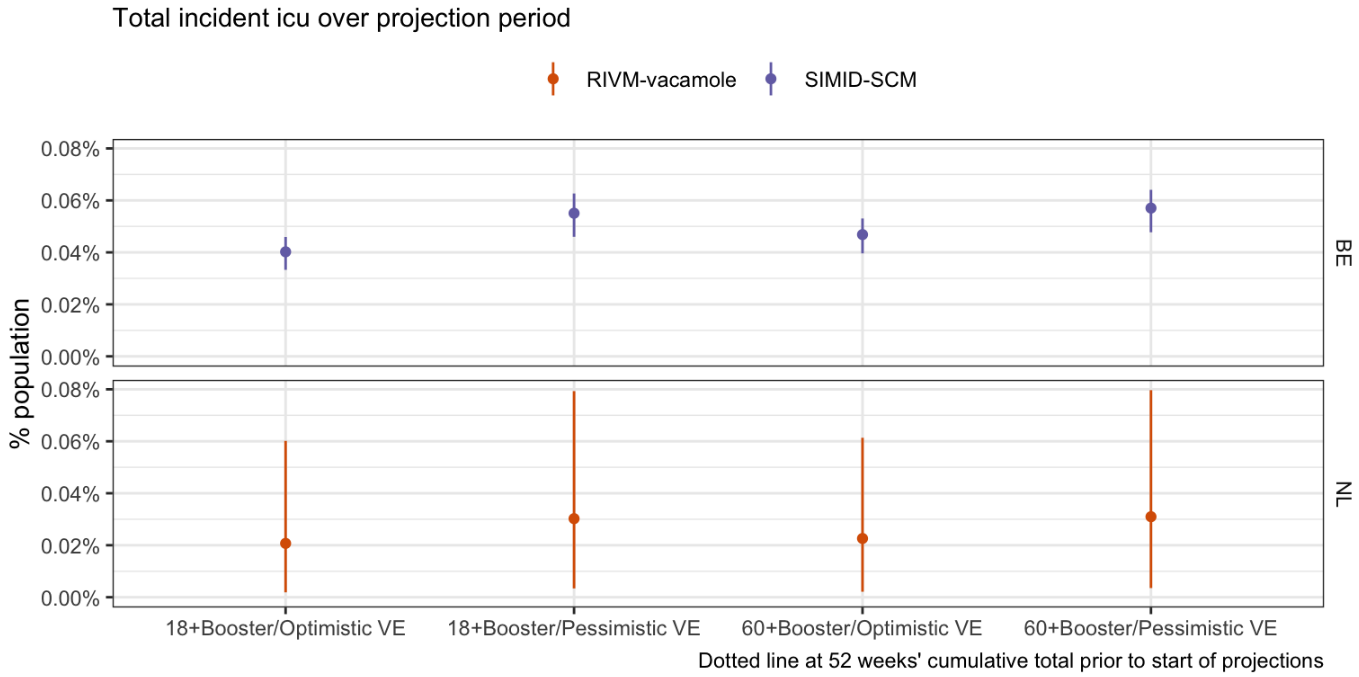

Scenarios: Autumn second booster campaign among population aged ‘18+’ or ‘60+’; Vaccine effectiveness is ‘optimistic’(effectiveness as of a booster vaccine against Delta) or ‘pessimistic’ (as against BA.4/BA.5/BA.2.75)

Incident outcomes

We explored the incidence of COVID-19 per 100,000 over the projection period and in terms of projected peaks in incidence. We summarised peaks both over the entire projection period, and over only the autumn-winter period (October through March); we considered (A) the timing and maximum weekly incidence of each peak, and (B) the total number of peaks.

Trajectories

Death

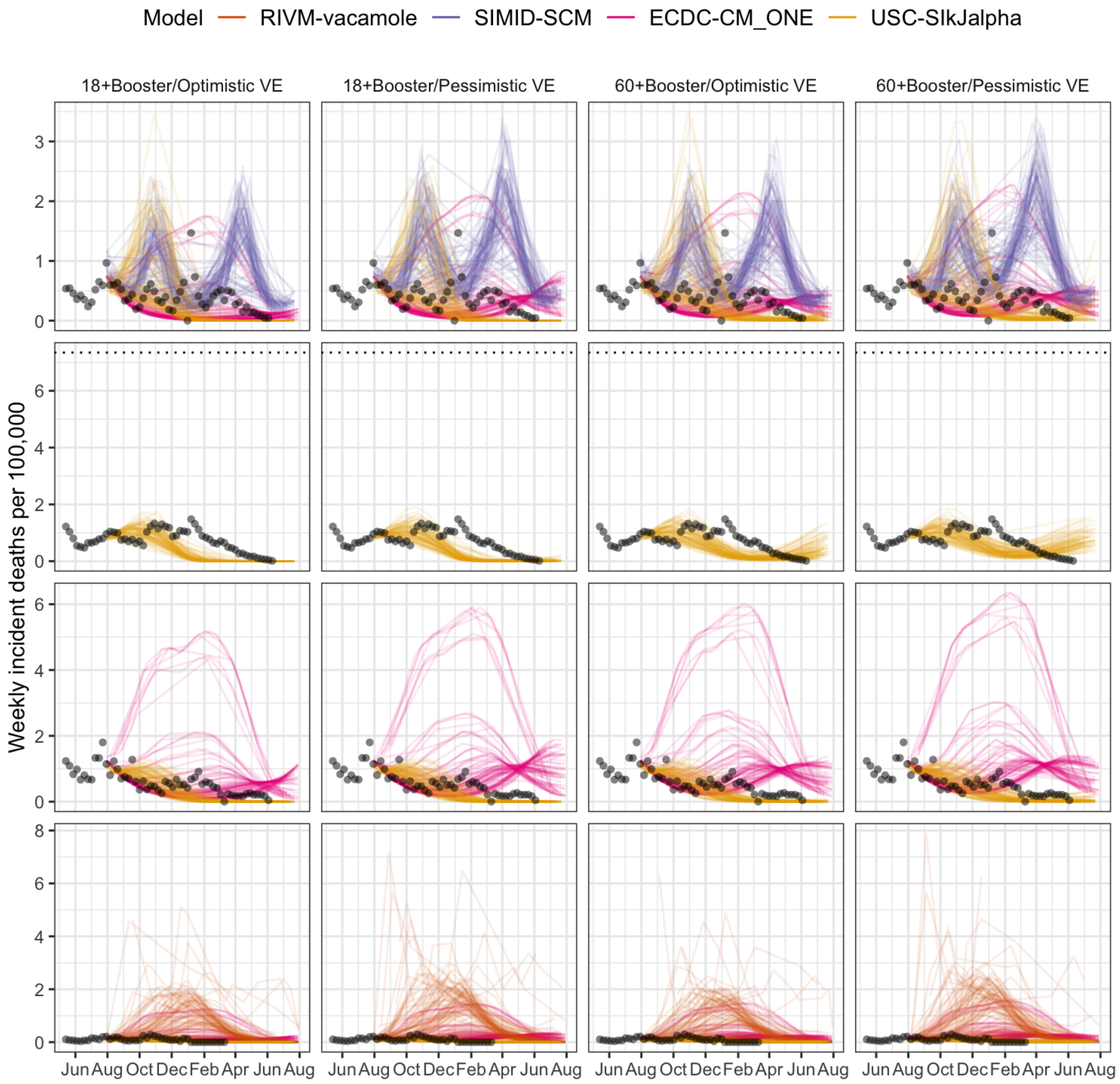

Scenarios: Autumn second booster campaign among population aged '18+' or '60+';
Vaccine effectiveness is 'optimistic'(effectiveness as of a booster vaccine against Delta) or 'pessimistic' (as against BA.4/BA.5/BA.2.75)

Case

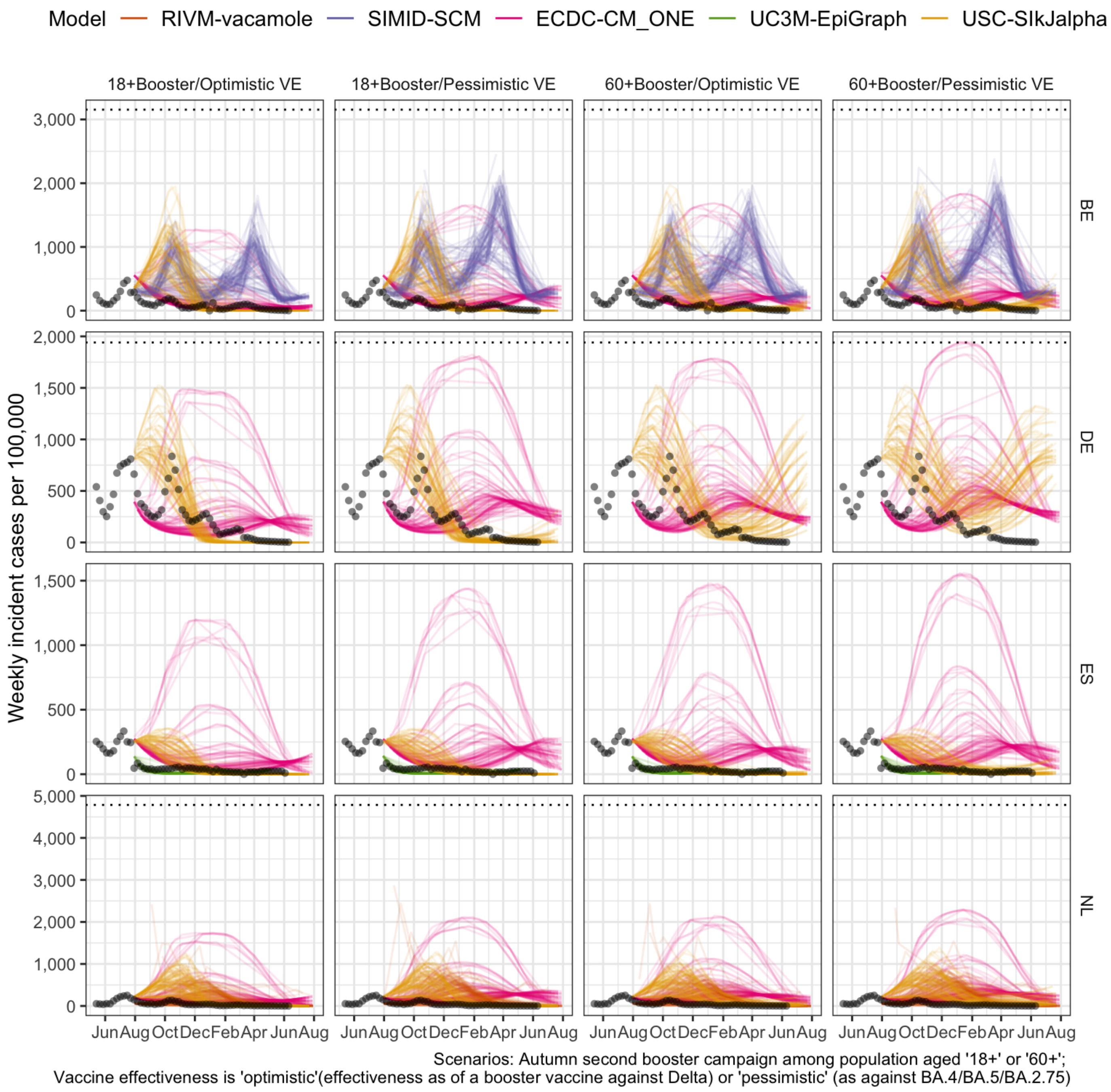

Model
RIVM-vacamole
SIMID-SCM
ECDC-CM_ONE
UC3M-EpiGraph
USC-SIkJalpha
18+Booster/Optimistic VE
18+Booster/Pessimistic VE
60+Booster/Optimistic VE
60+Booster/Pessimistic VE
BE
DE
ES
NL
Weekly incident cases per 100,000
3,000
2,000
1,000
0
2,000
1,500
1,000
500
0
1,500
1,000
500
0
5,000
4,000
3,000
2,000
1,000
0
Jun Aug Oct Dec Feb Apr Jun Aug
Scenarios: Autumn second booster campaign among population aged '18+' or '60+';
Vaccine effectiveness is 'optimistic'(effectiveness as of a booster vaccine against Delta) or 'pessimistic' (as against BA.4/BA.5/BA.2.75)

Infection

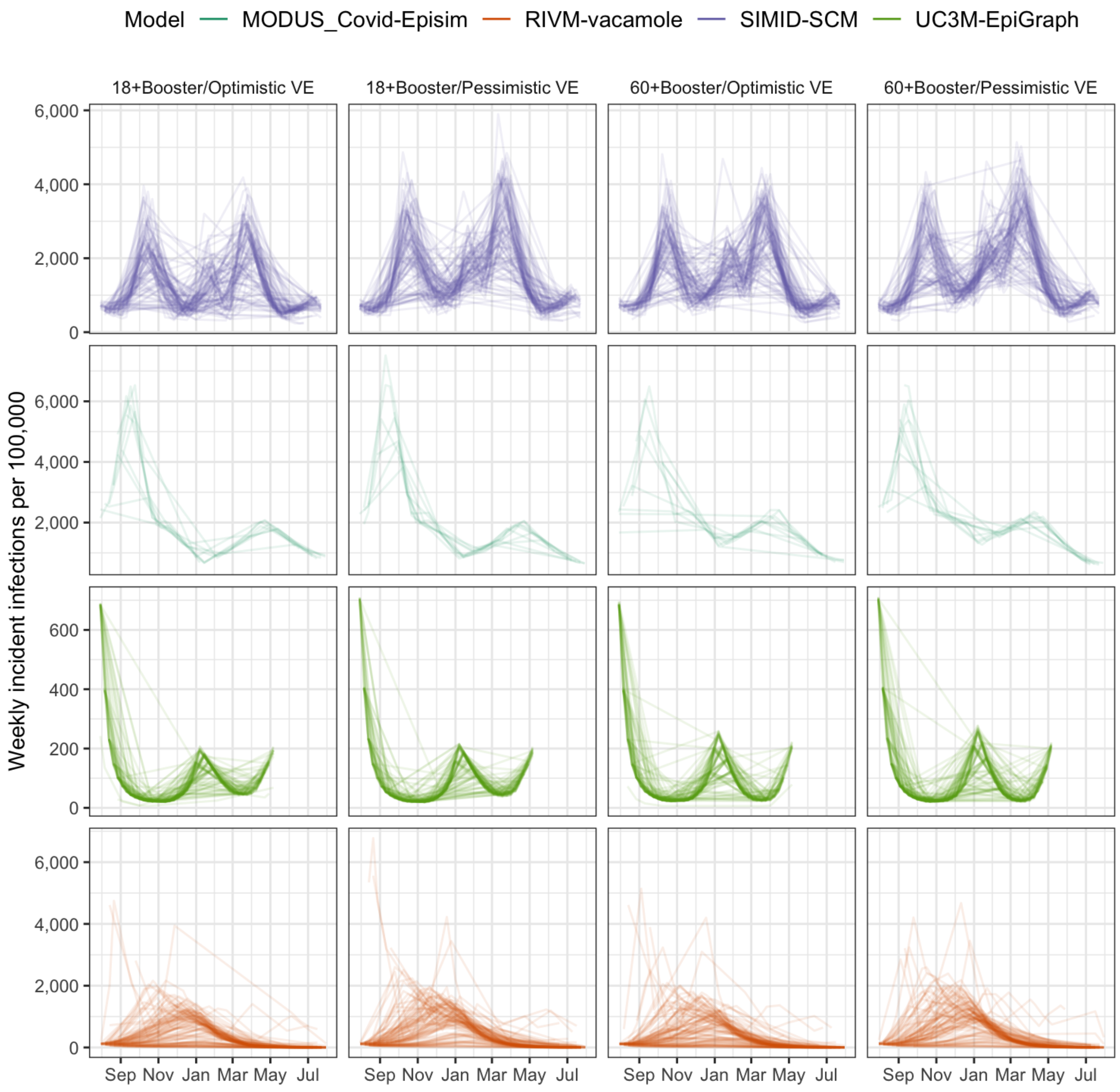

Scenarios: Autumn second booster campaign among population aged '18+' or '60+';
Vaccine effectiveness is 'optimistic'(effectiveness as of a booster vaccine against Delta) or 'pessimistic' (as against BA.4/BA.5/BA.2.75)

Hosp

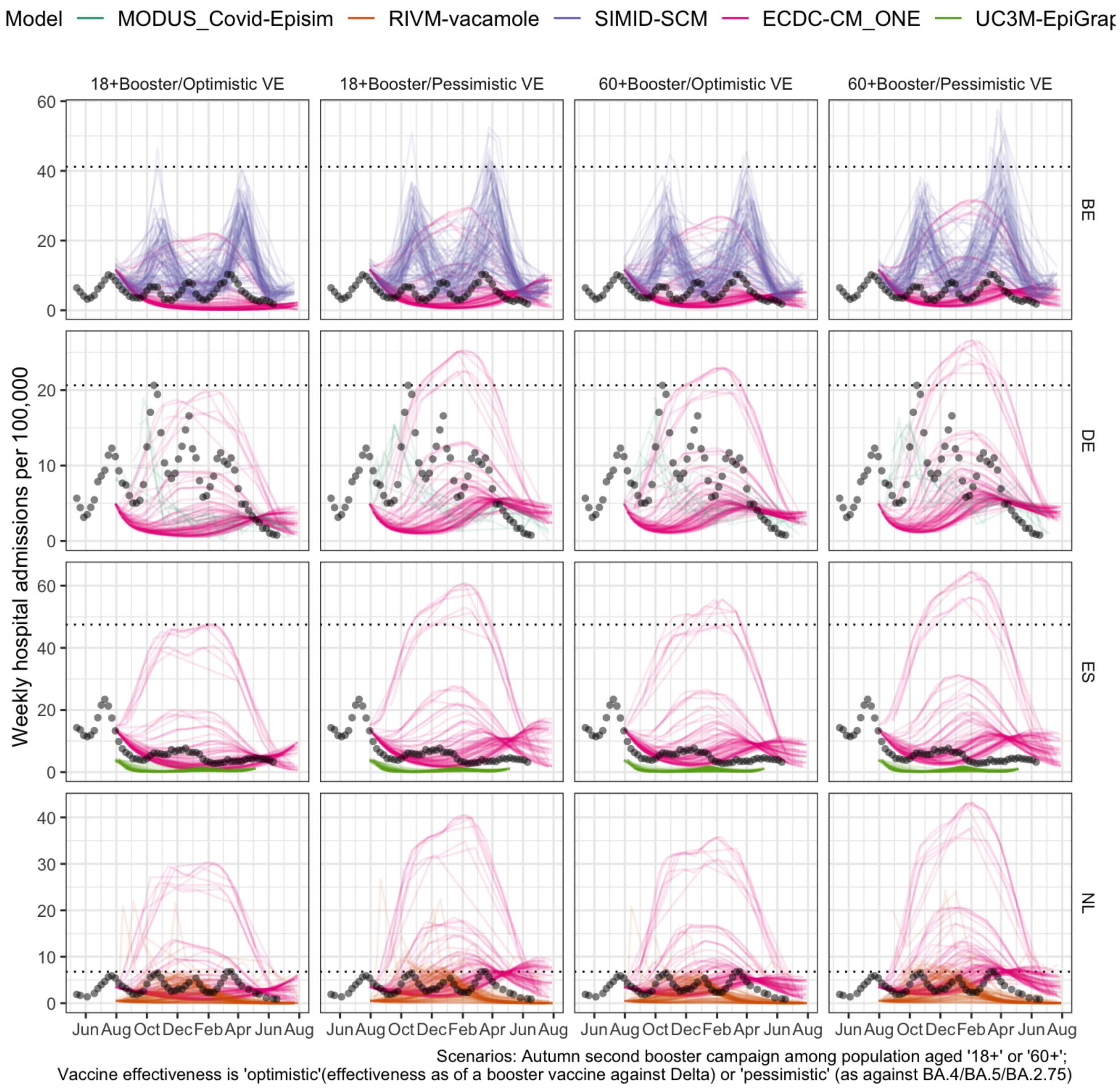

Model
MODUS_Covid-Episim
RIVM-vacamole
SIMID-SCM
ECDC-CM_ONE
UC3M-EpiGrap
18+Booster/Optimistic VE
18+Booster/Pessimistic VE
60+Booster/Optimistic VE
60+Booster/Pessimistic VE
BE
DE
ES
NL
Weekly hospital admissions per 100,000
Jun Aug Oct Dec Feb Apr Jun Aug
Scenarios: Autumn second booster campaign among population aged '18+' or '60+';
Vaccine effectiveness is 'optimistic'(effectiveness as of a booster vaccine against Delta) or 'pessimistic' (as against BA.4/BA.5/BA.2.75)

Icu

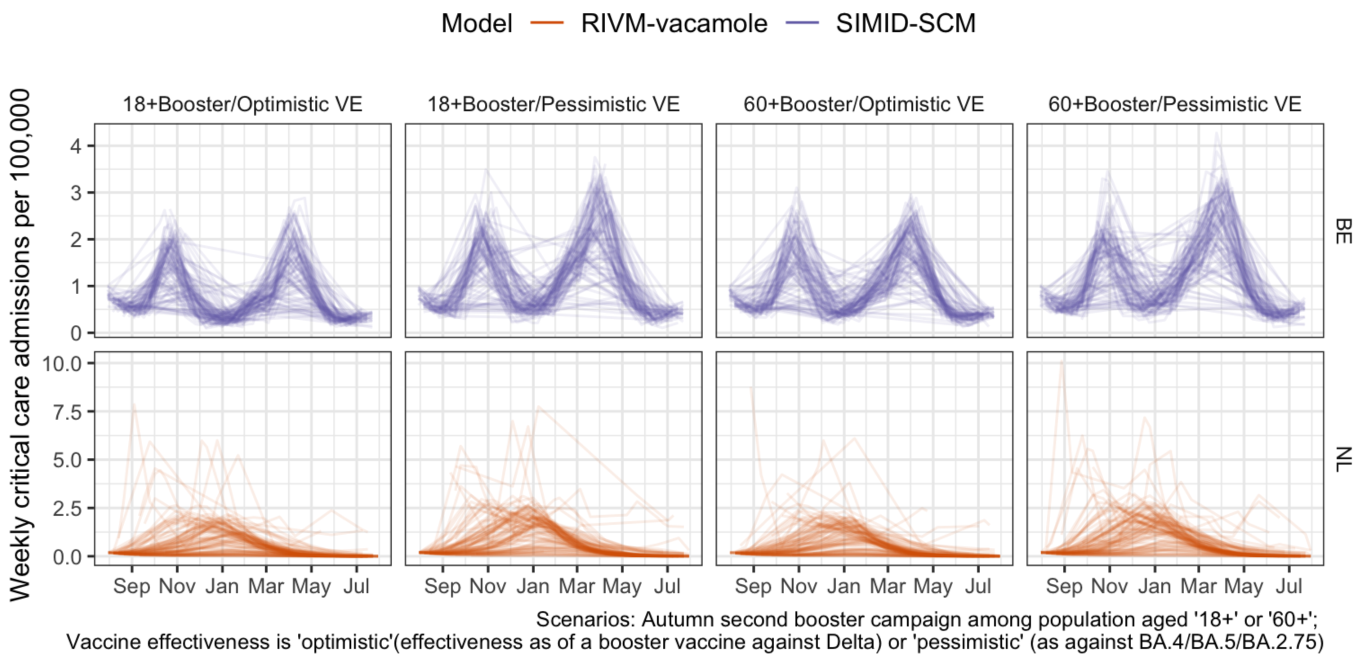

Peaks

Autumn-winter

Projections over October 2022 through March 2023

Death

A. Size and timing of peaks. Boxplots show summary of the likely value at peak incidence (median and interquartile range); points show timing and size of peaks from independent sample simulations

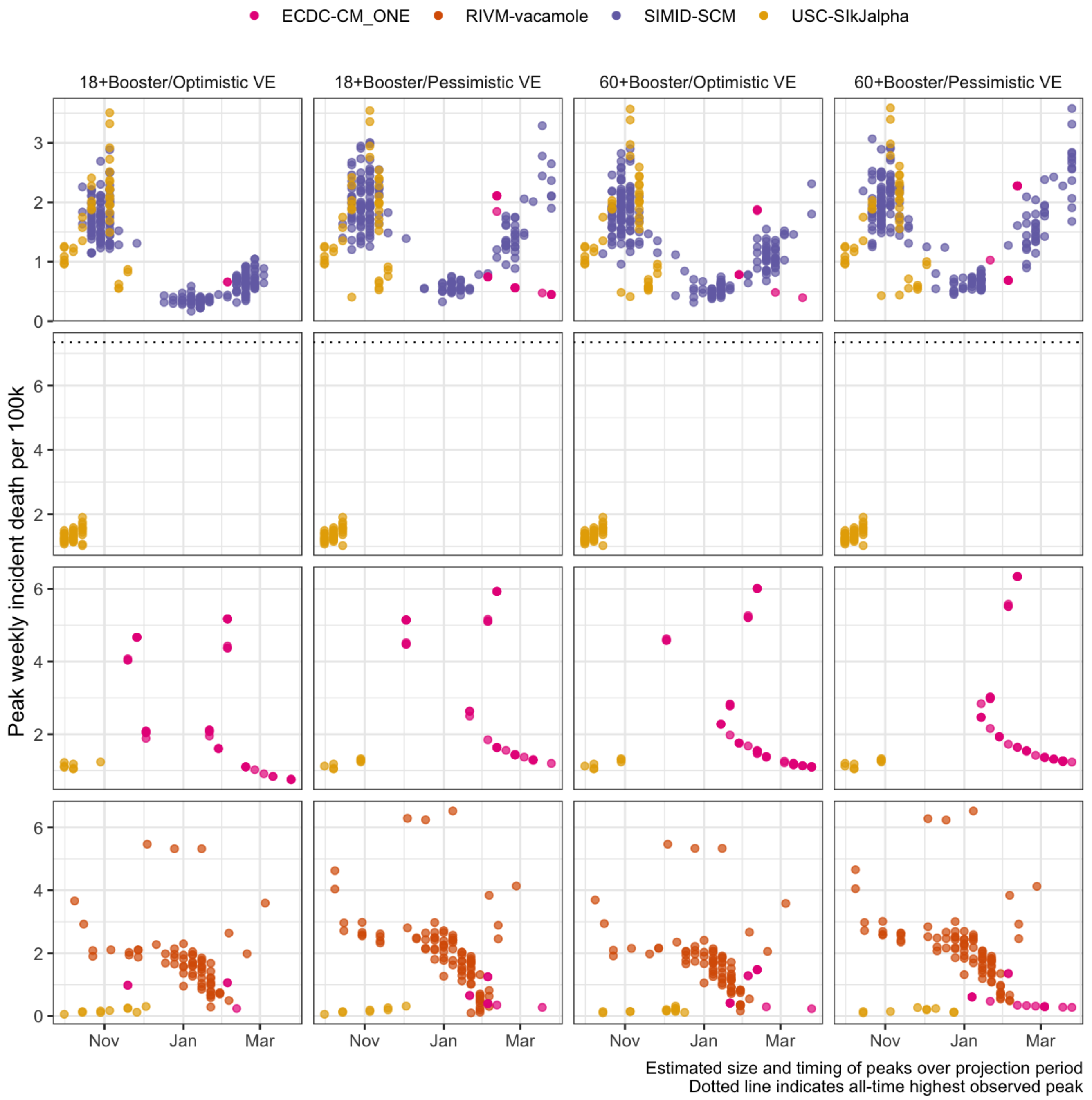

B. Projected number of peaks (median with 5-95% probability)

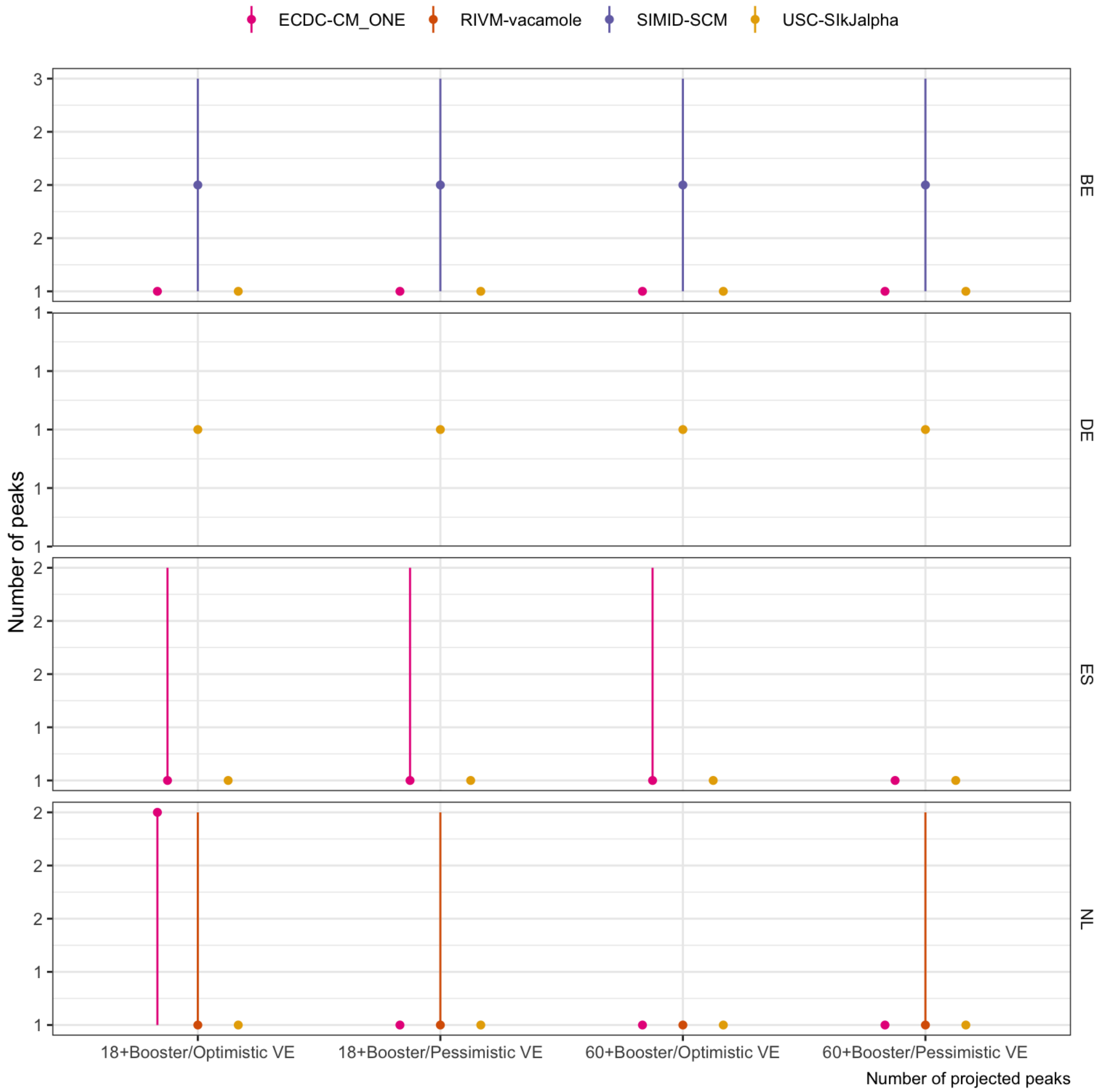

Scenarios: Autumn second booster campaign among population aged ‘18+’ or ‘60+’; Vaccine effectiveness is ‘optimistic’(effectiveness as of a booster vaccine against Delta) or ‘pessimistic’ (as against BA.4/BA.5/BA.2.75)

Case

A. Size and timing of peaks. Boxplots show summary of the likely value at peak incidence (median and interquartile range); points show timing and size of peaks from independent sample simulations

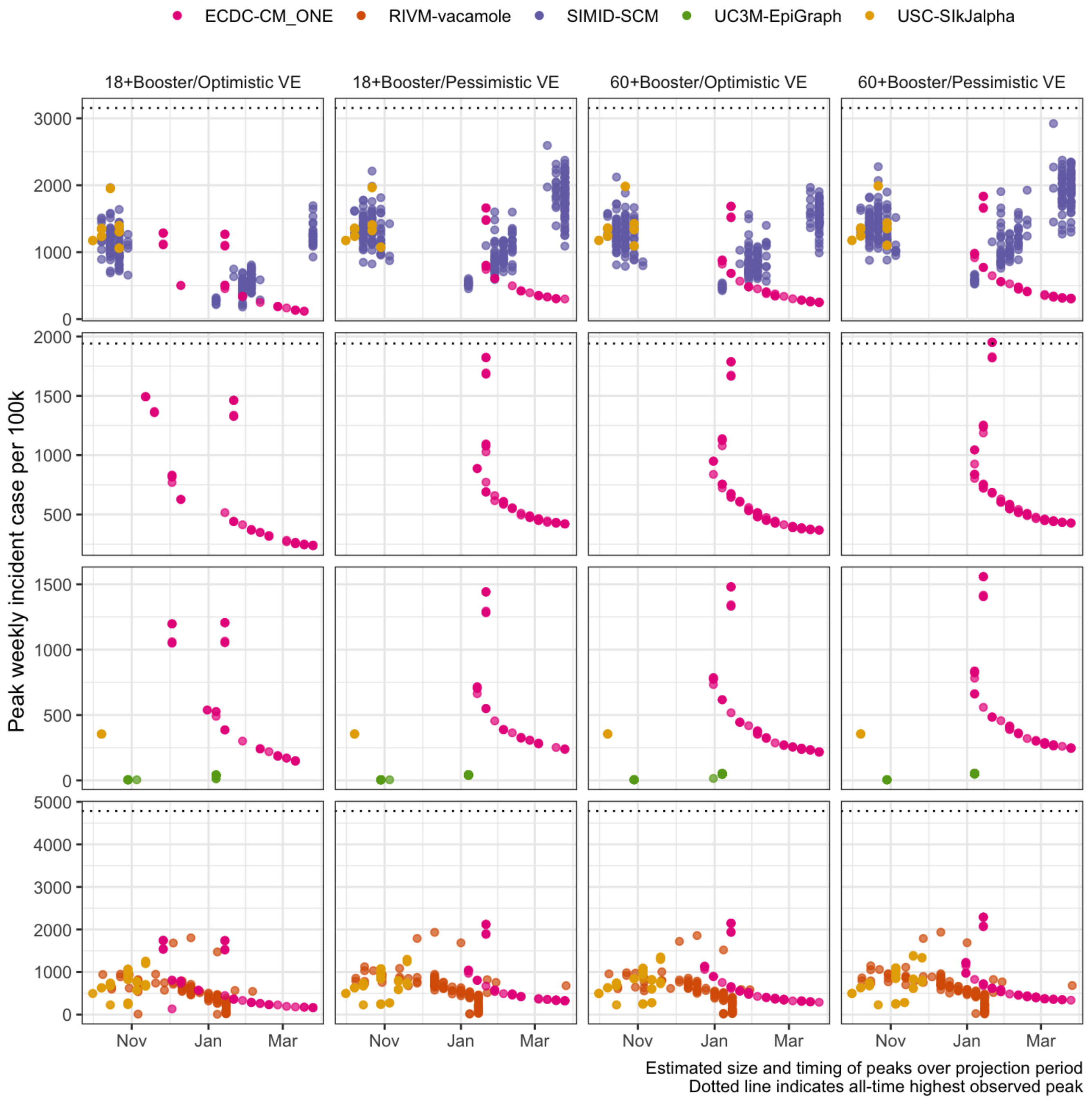

B. Projected number of peaks (median with 5-95% probability)

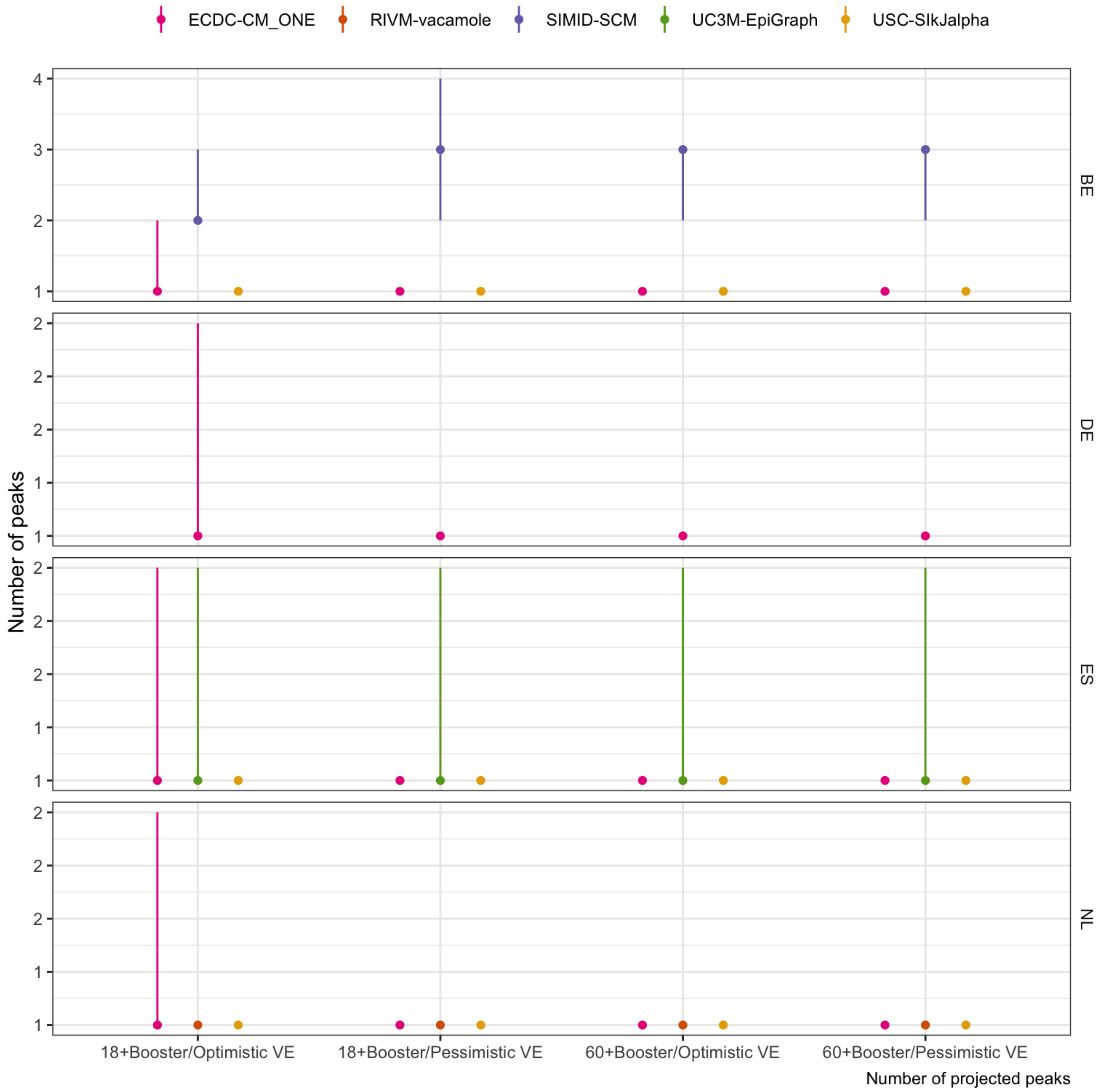

Scenarios: Autumn second booster campaign among population aged ‘18+’ or ‘60+’; Vaccine effectiveness is ‘optimistic’(effectiveness as of a booster vaccine against Delta) or ‘pessimistic’ (as against BA.4/BA.5/BA.2.75)

Infection

A. Size and timing of peaks. Boxplots show summary of the likely value at peak incidence (median and interquartile range); points show timing and size of peaks from independent sample simulations

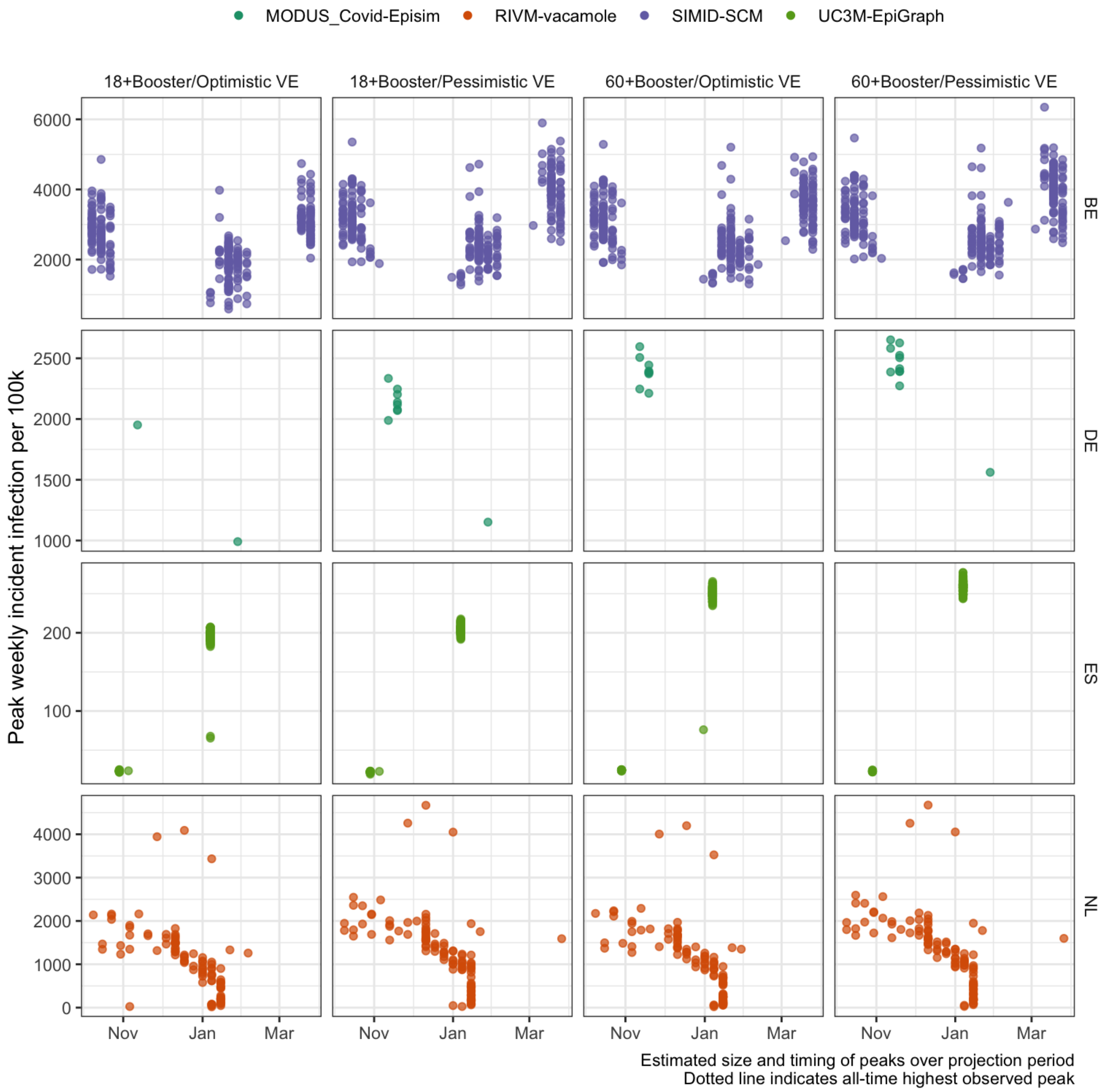

B. Projected number of peaks (median with 5-95% probability)

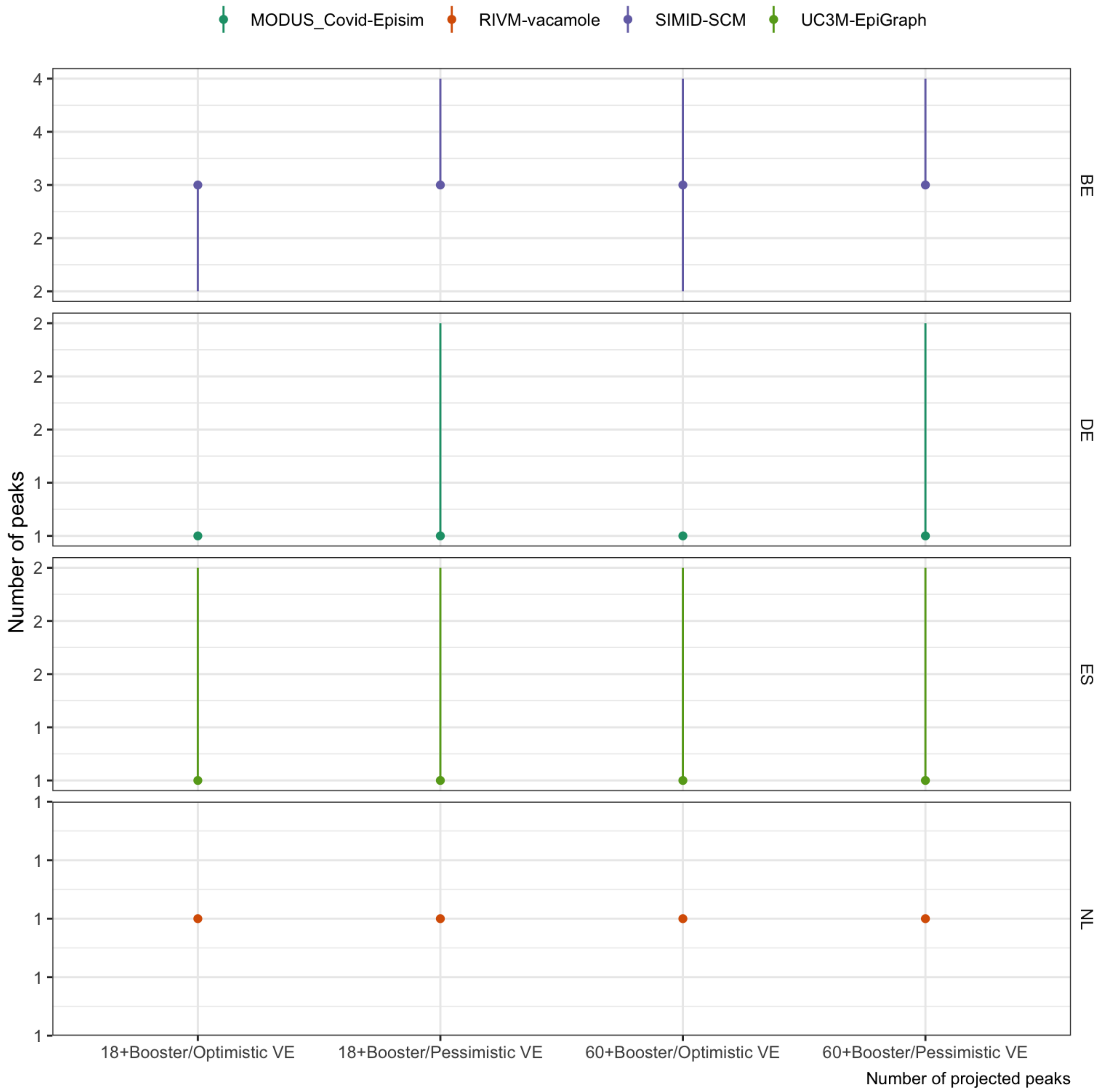

Scenarios: Autumn second booster campaign among population aged ‘18+’ or ‘60+’; Vaccine effectiveness is ‘optimistic’(effectiveness as of a booster vaccine against Delta) or ‘pessimistic’ (as against BA.4/BA.5/BA.2.75)

Hosp

A. Size and timing of peaks. Boxplots show summary of the likely value at peak incidence (median and interquartile range); points show timing and size of peaks from independent sample simulations

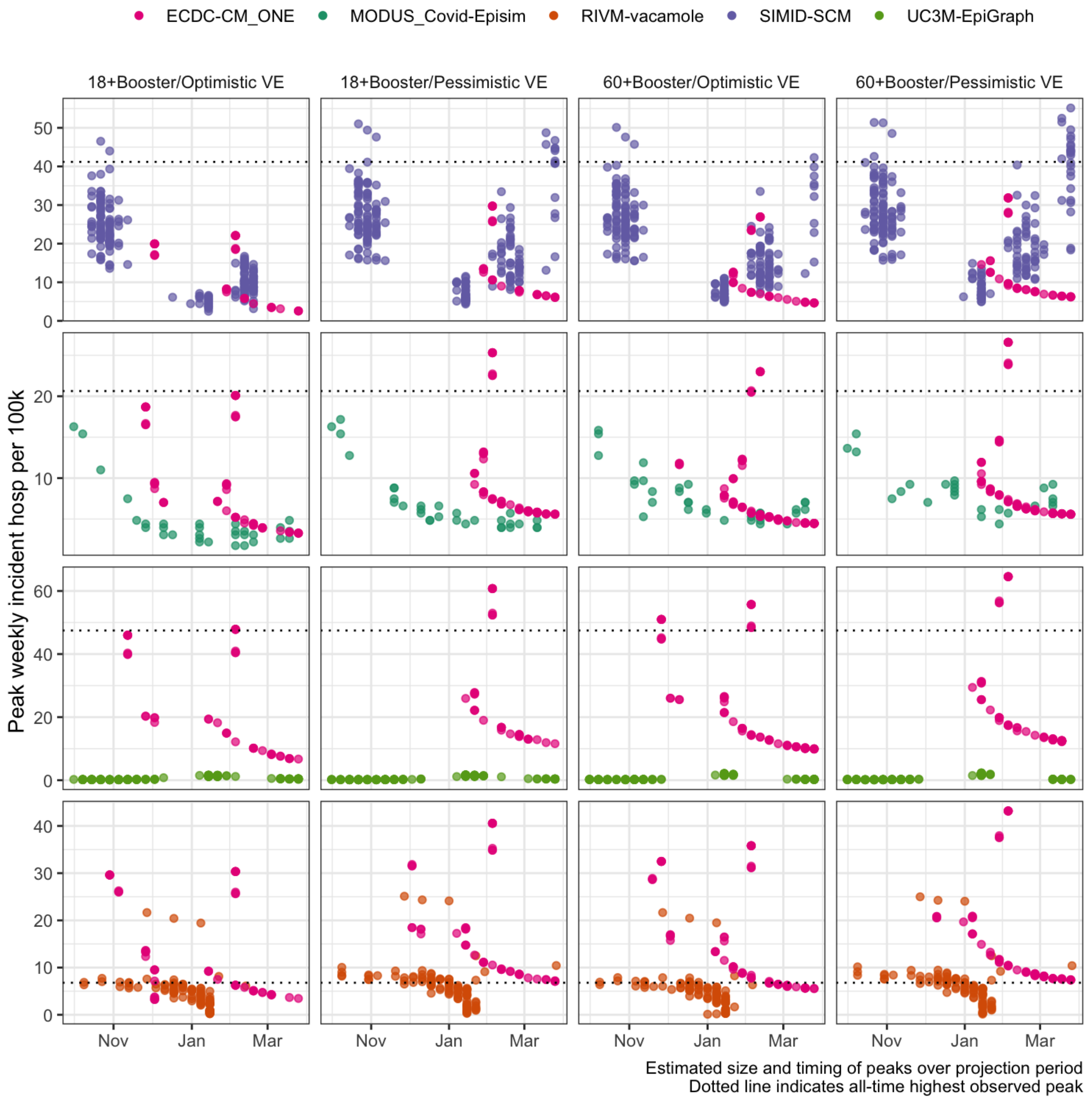

B. Projected number of peaks (median with 5-95% probability)

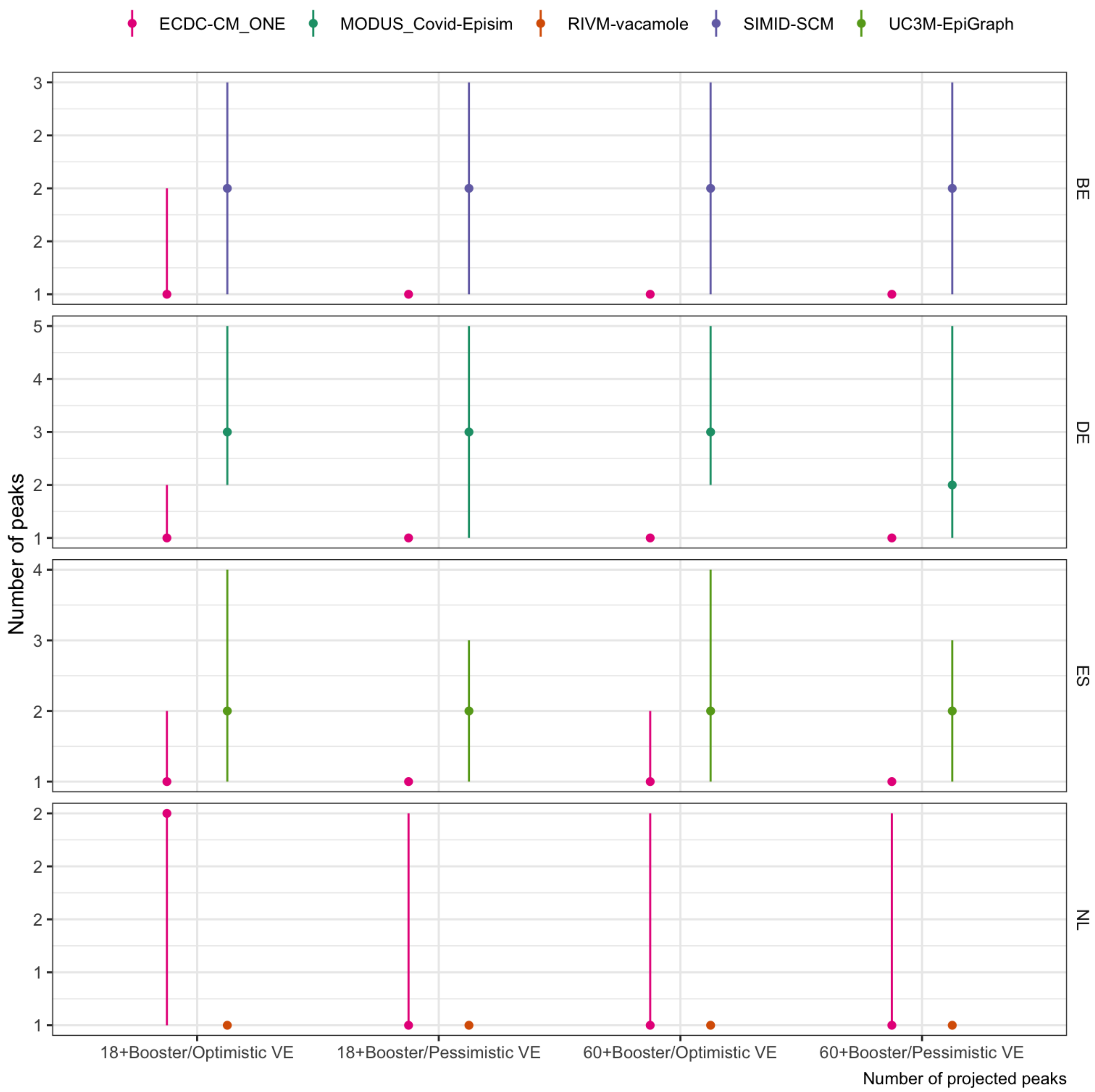

Scenarios: Autumn second booster campaign among population aged ‘18+’ or ‘60+’; Vaccine effectiveness is ‘optimistic’(effectiveness as of a booster vaccine against Delta) or ‘pessimistic’ (as against BA.4/BA.5/BA.2.75)

Icu

A. Size and timing of peaks. Boxplots show summary of the likely value at peak incidence (median and interquartile range); points show timing and size of peaks from independent sample simulations

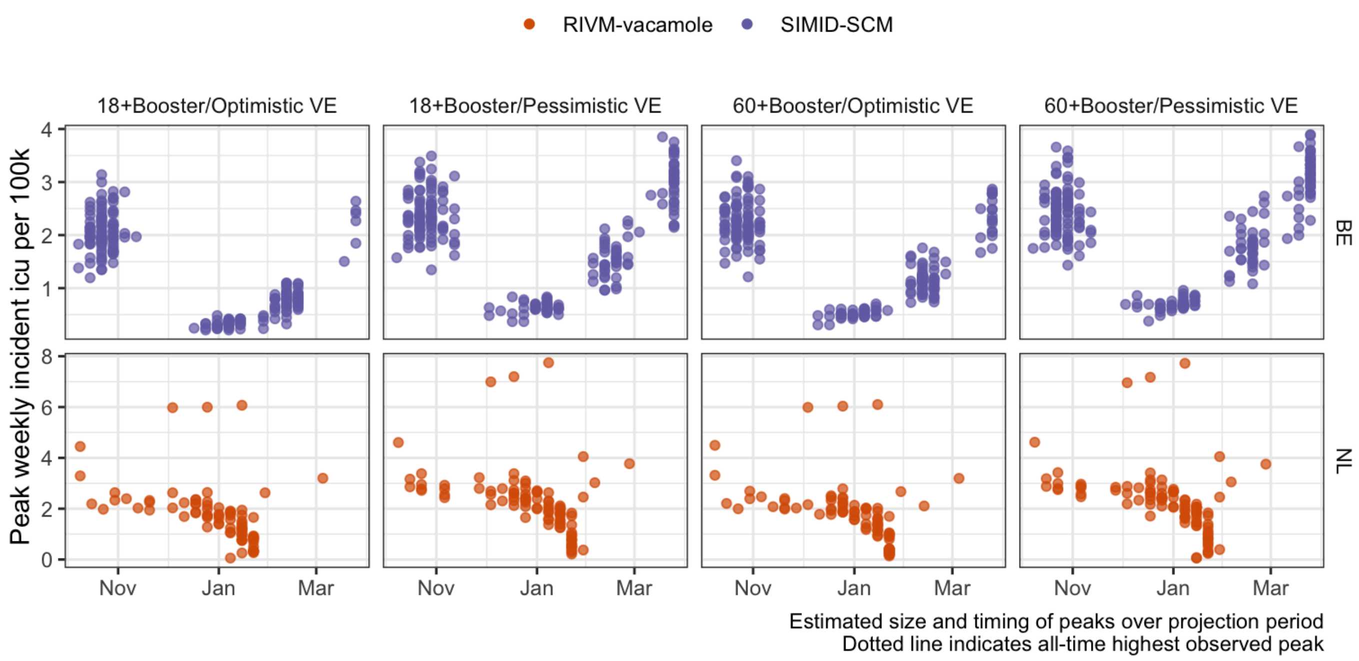

B. Projected number of peaks (median with 5-95% probability)

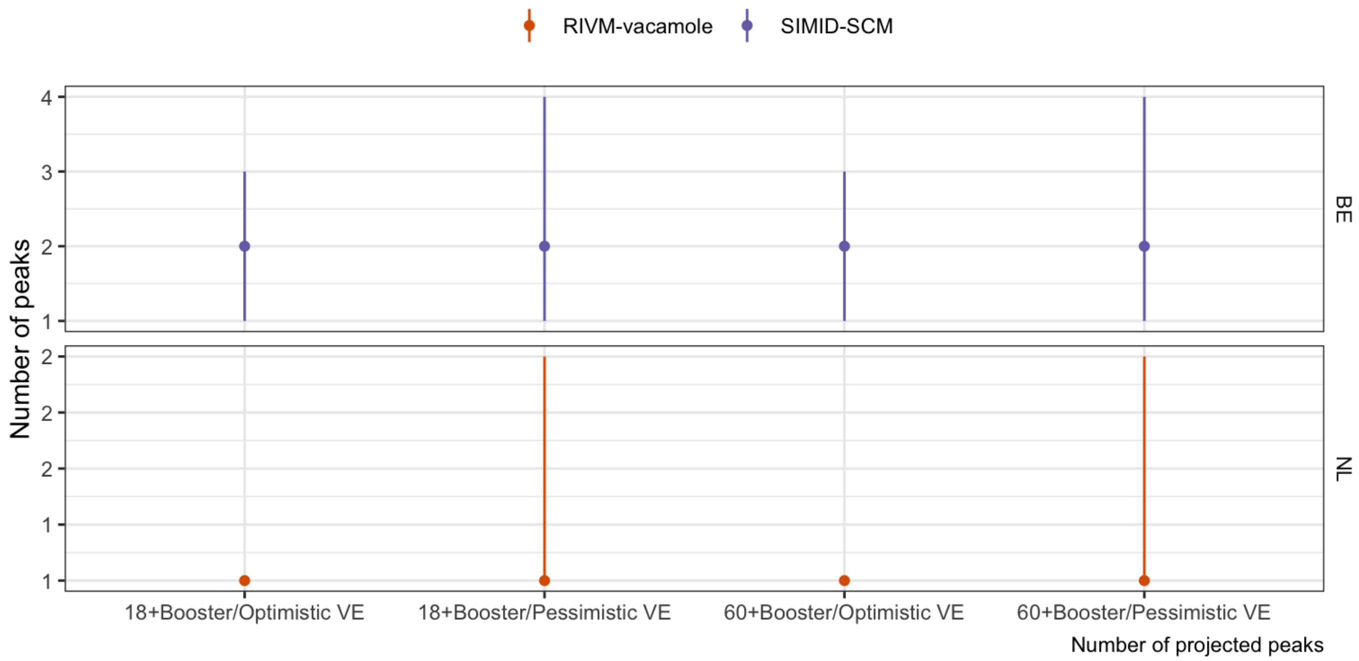

Scenarios: Autumn second booster campaign among population aged '18+' or '60+'; Vaccine effectiveness is 'optimistic'(effectiveness as of a booster vaccine against Delta) or 'pessimistic' (as against BA.4/BA.5/BA.2.75)

Entire projection period

Projections over June 2022 through June 2023

Death

A. Size and timing of peaks. Boxplots show summary of the likely value at peak incidence (median and interquartile range); points show timing and size of peaks from independent sample simulations

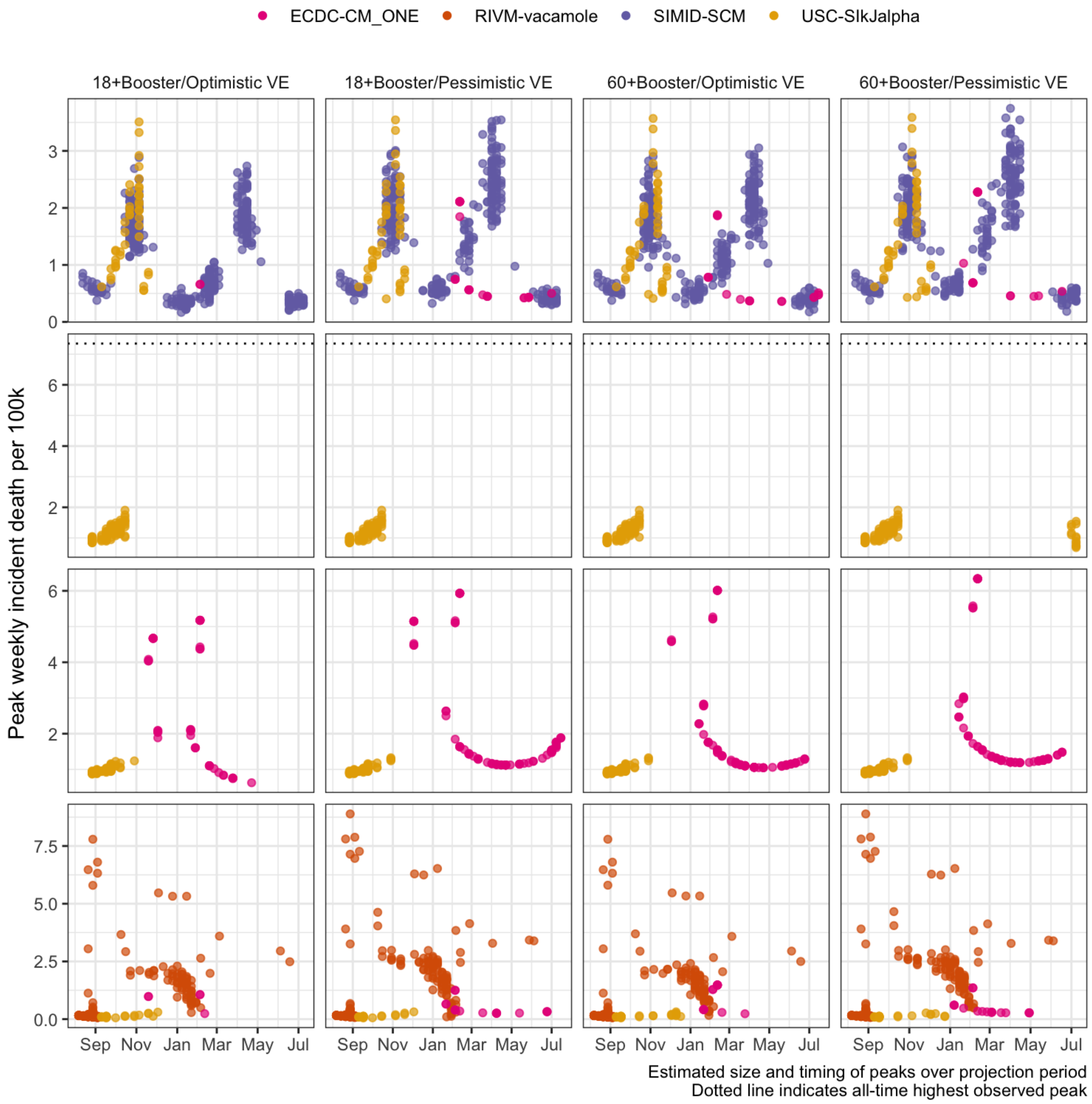

B. Projected number of peaks (median with 5-95% probability)

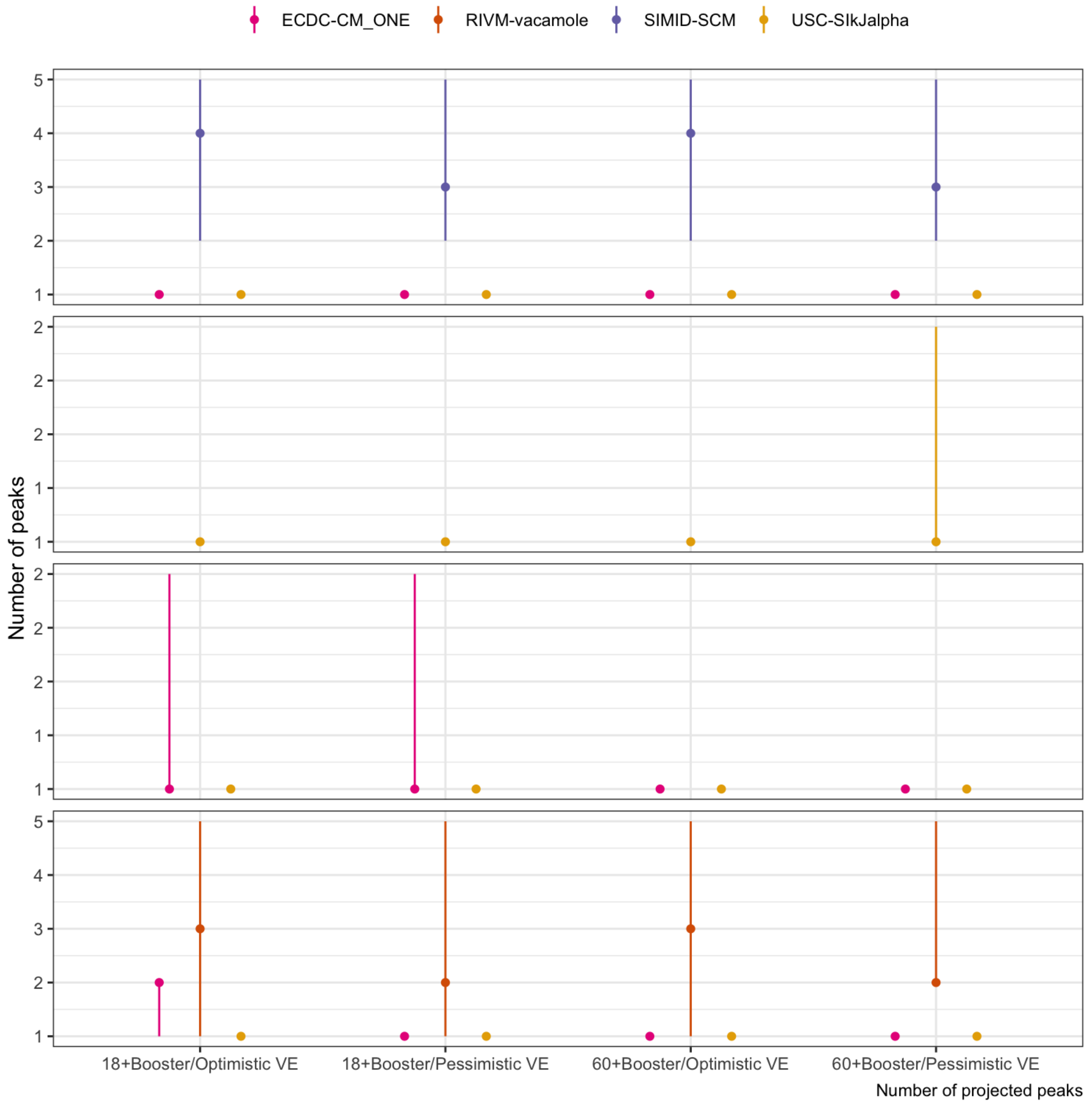

Scenarios: Autumn second booster campaign among population aged ‘18+’ or ‘60+’; Vaccine effectiveness is ‘optimistic’(effectiveness as of a booster vaccine against Delta) or ‘pessimistic’ (as against BA.4/BA.5/BA.2.75)

Case

A. Size and timing of peaks. Boxplots show summary of the likely value at peak incidence (median and interquartile range); points show timing and size of peaks from independent sample simulations

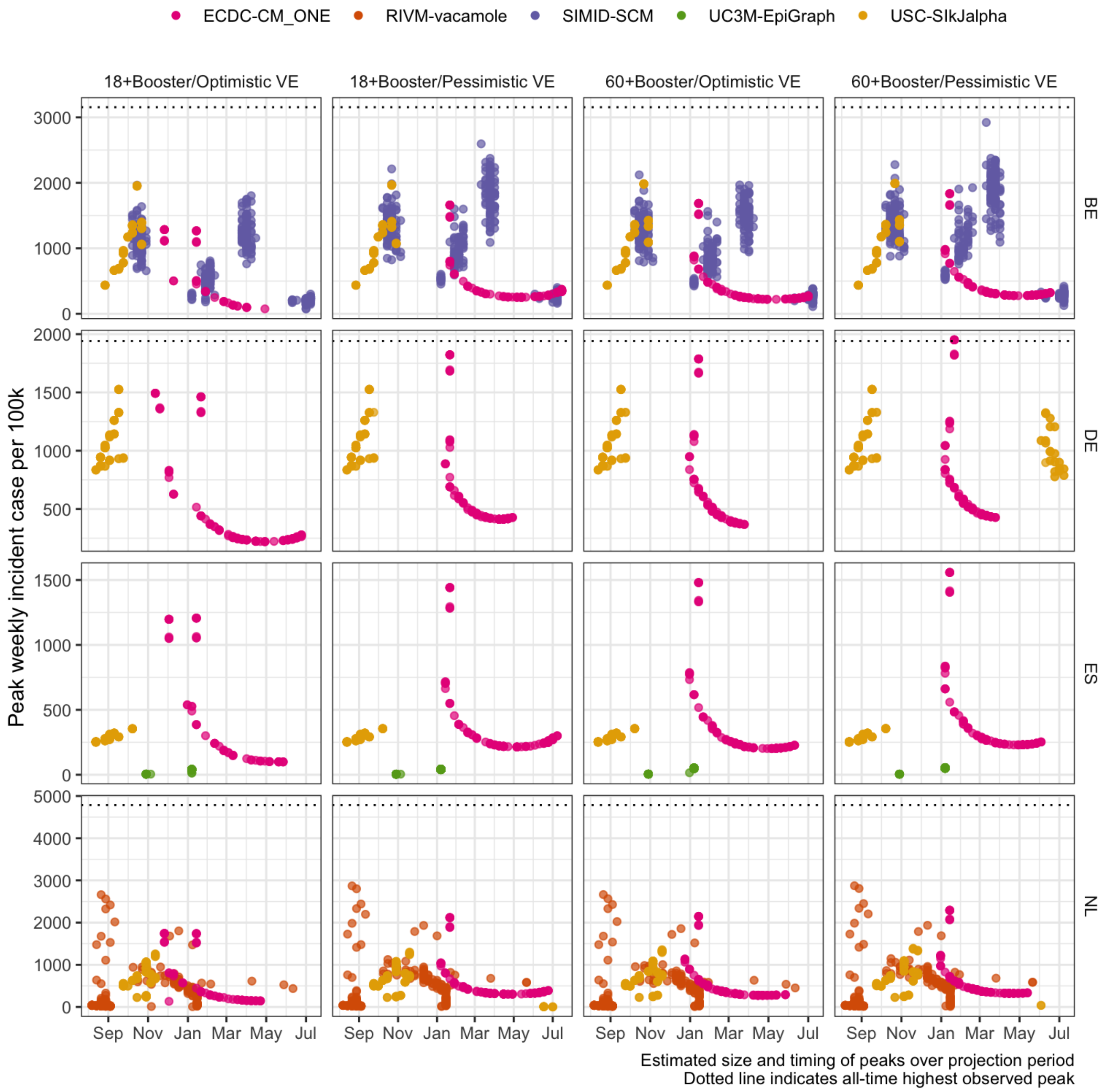

B. Projected number of peaks (median with 5-95% probability)

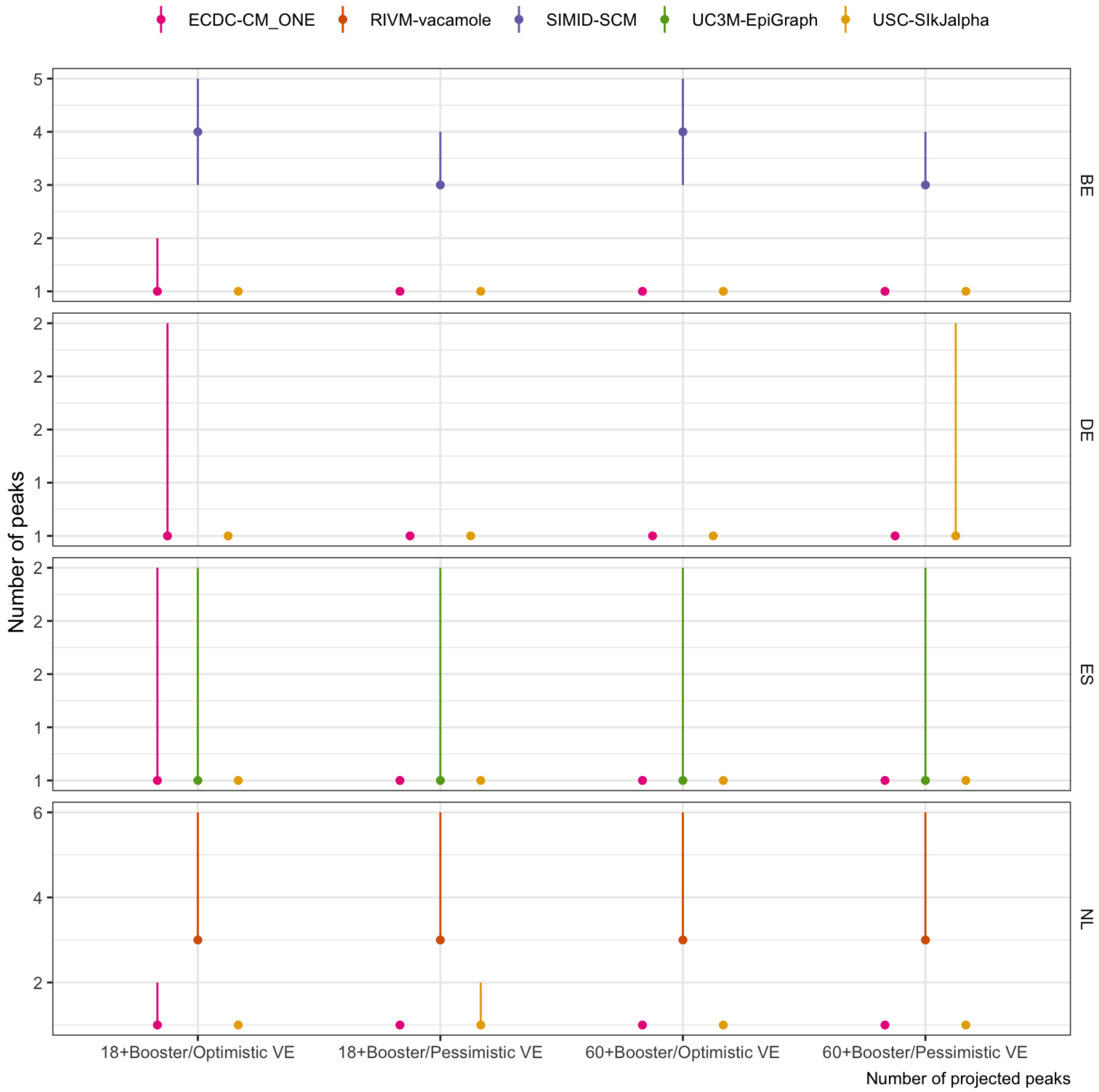

Scenarios: Autumn second booster campaign among population aged ‘18+’ or ‘60+’; Vaccine effectiveness is ‘optimistic’(effectiveness as of a booster vaccine against Delta) or ‘pessimistic’ (as against BA.4/BA.5/BA.2.75)

Infection

A. Size and timing of peaks. Boxplots show summary of the likely value at peak incidence (median and interquartile range); points show timing and size of peaks from independent sample simulations

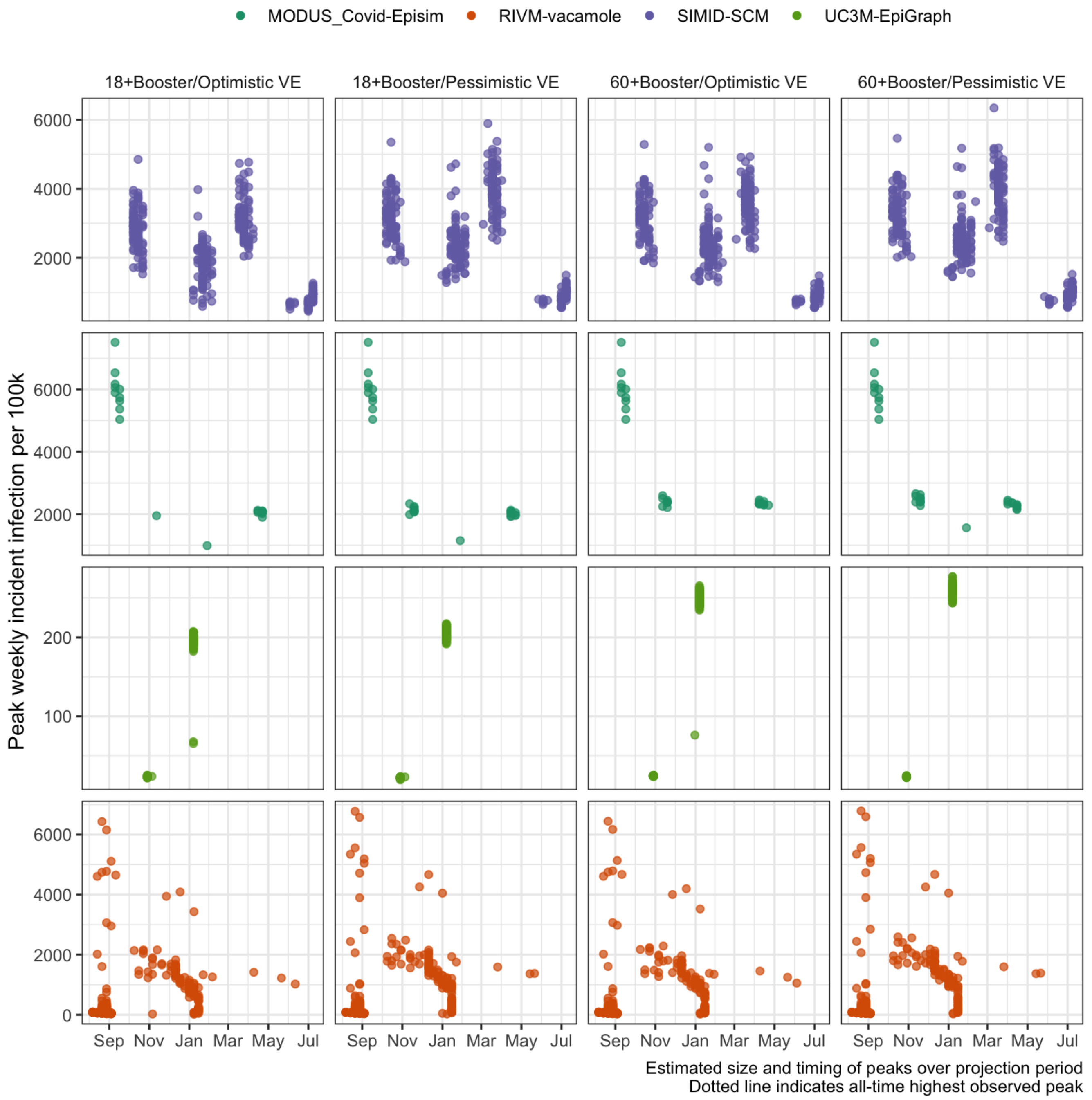

B. Projected number of peaks (median with 5-95% probability)

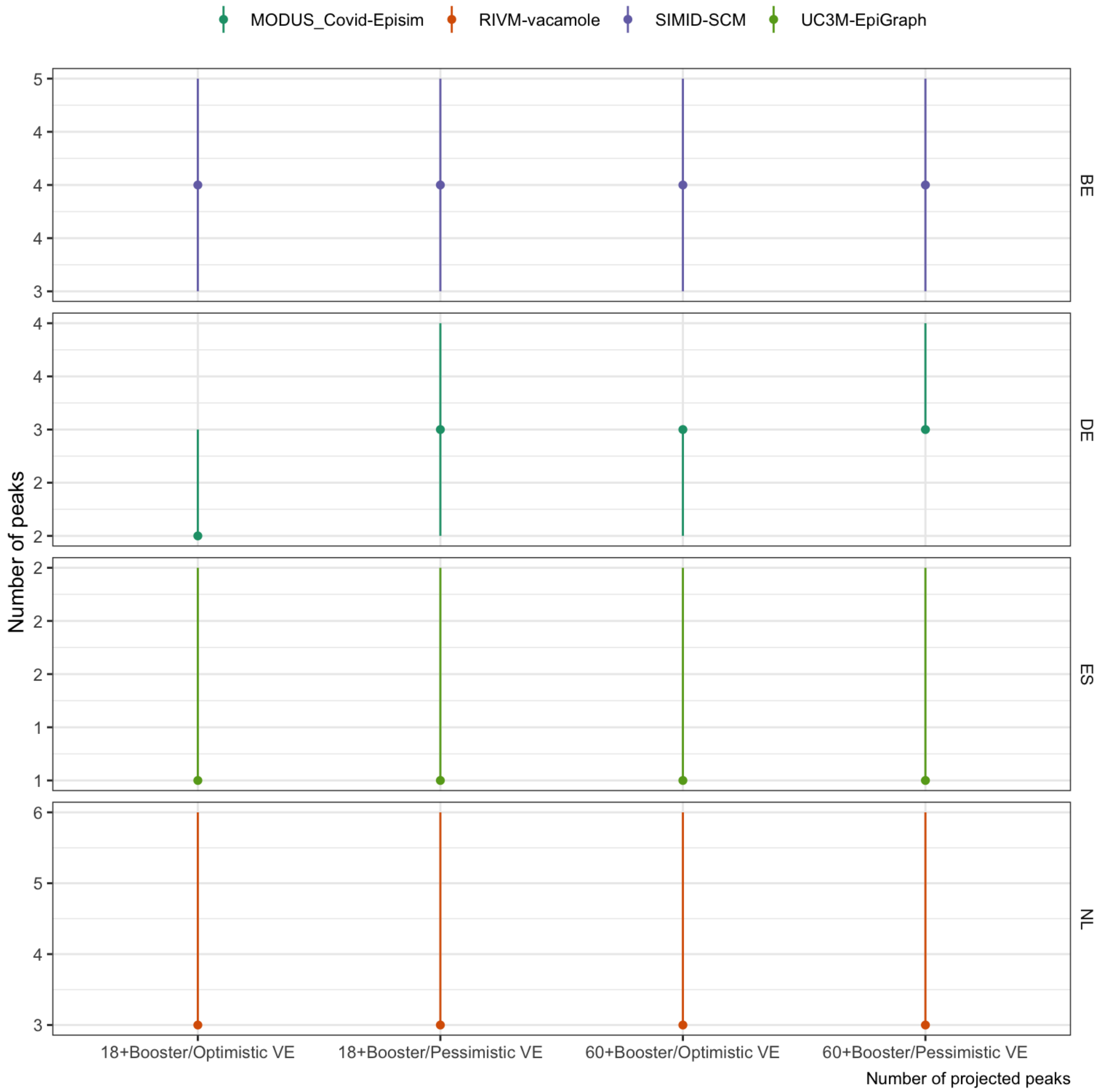

Scenarios: Autumn second booster campaign among population aged ‘18+’ or ‘60+’; Vaccine effectiveness is ‘optimistic’(effectiveness as of a booster vaccine against Delta) or ‘pessimistic’ (as against BA.4/BA.5/BA.2.75)

Hosp

A. Size and timing of peaks. Boxplots show summary of the likely value at peak incidence (median and interquartile range); points show timing and size of peaks from independent sample simulations

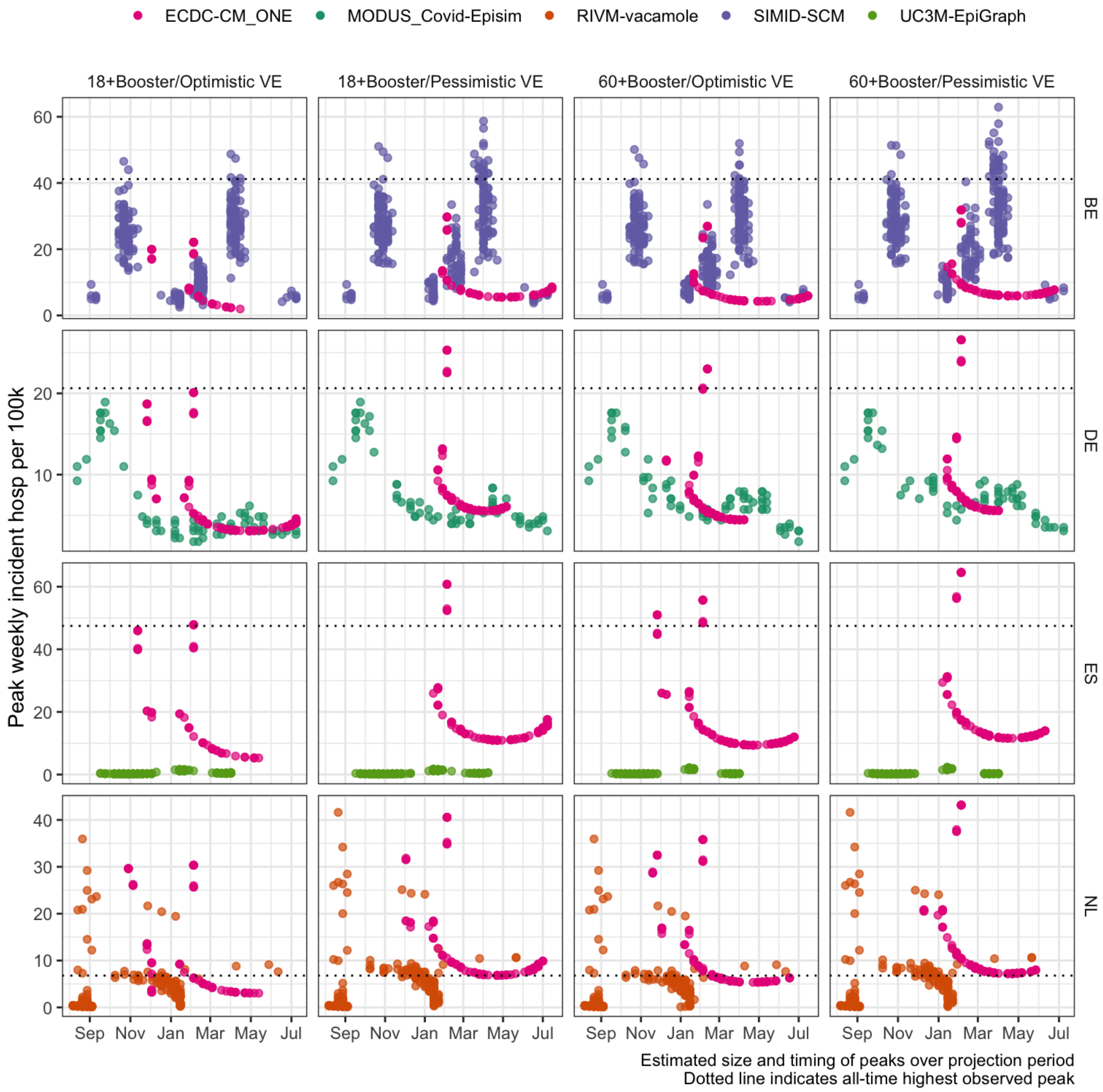

B. Projected number of peaks (median with 5-95% probability)

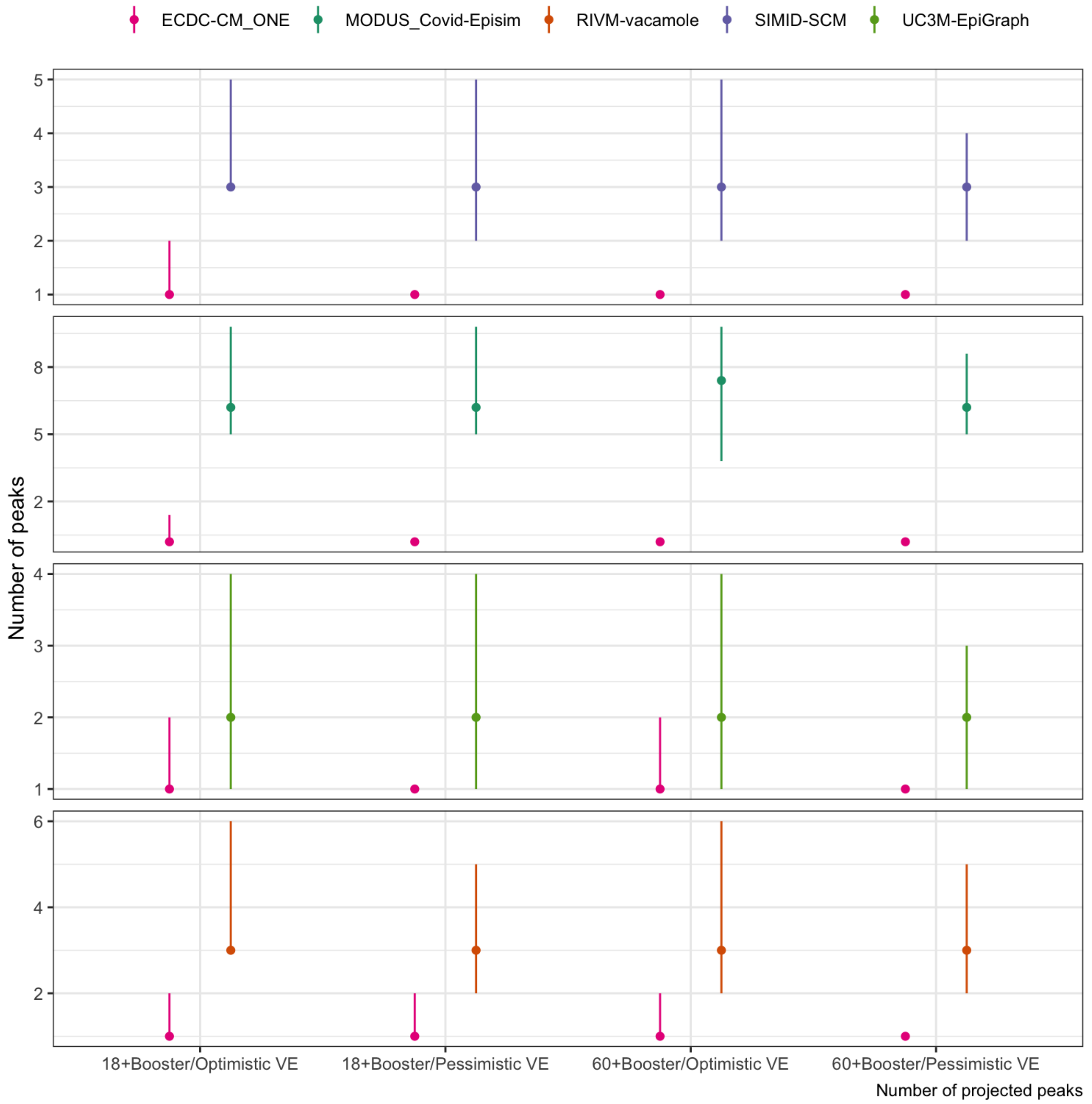

Scenarios: Autumn second booster campaign among population aged ‘18+’ or ‘60+’; Vaccine effectiveness is ‘optimistic’(effectiveness as of a booster vaccine against Delta) or ‘pessimistic’ (as against BA.4/BA.5/BA.2.75)

Icu

A. Size and timing of peaks. Boxplots show summary of the likely value at peak incidence (median and interquartile range); points show timing and size of peaks from independent sample simulations

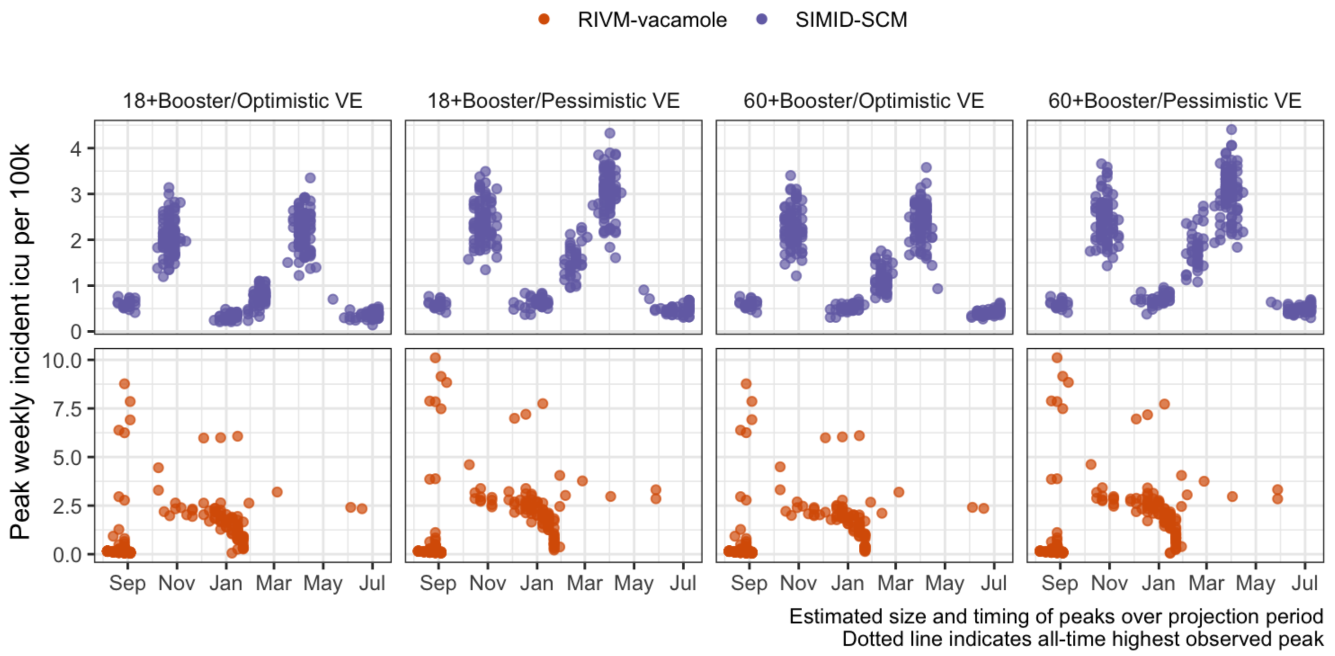

B. Projected number of peaks (median with 5-95% probability)

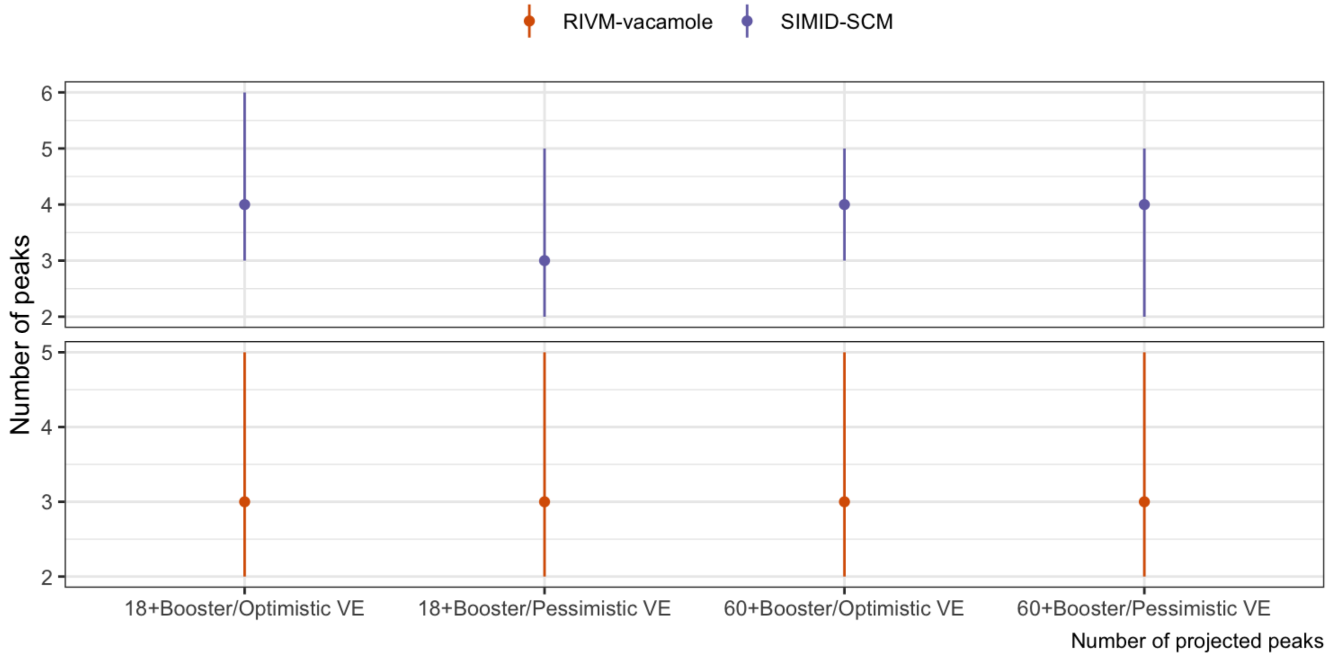

Scenarios: Autumn second booster campaign among population aged '18+' or '60+'; Vaccine effectiveness is 'optimistic'(effectiveness as of a booster vaccine against Delta) or 'pessimistic' (as against BA.4/BA.5/BA.2.75)

The European Scenario and Forecast (https://covid19forecasthub.eu/index.html) Hubs are run in collaboration between the Epiforecasts team (https://epiforecasts.io/) at the London School of Hygiene & Tropical Medicine (https://www.lshtm.ac.uk); and the European Centre for Disease Control and Prevention (ECDC) (https://ecdc.europa.eu).

Contact us (contact.html)
